# Development of a Specific Aptamer‐Modified Nano‐System to Treat Esophageal Squamous Cell Carcinoma

**DOI:** 10.1002/advs.202309084

**Published:** 2024-05-05

**Authors:** Fei Xie, Jinrong Qiu, Congyong Sun, Lulu Feng, Yali Jun, Chao Luo, Xiamei Guo, Bowei Zhang, Yu Zhou, Yuting Wang, Li Zhang, Qilong Wang

**Affiliations:** ^1^ The Comprehensive Cancer Center, Department of Central Laboratory, The Affiliated Huaian No.1 People's Hospital Nanjing Medical University Huai'an Jiangsu 223300 China; ^2^ The Comprehensive Cancer Center, Department of Clinical Oncology, The Affiliated Huaian No.1 People's Hospital Nanjing Medical University Huai'an Jiangsu 223300 China

**Keywords:** DNA aptamers, drug delivery, esophageal squamous cell carcinoma, nano‐systems, tumor targeting

## Abstract

Esophageal squamous cell carcinoma (ESCC) is a prevalent gastrointestinal cancer characterized by high mortality and an unfavorable prognosis. While combination therapies involving surgery, chemotherapy, and radiation therapy are advancing, targeted therapy for ESCC remains underdeveloped. As a result, the overall five‐year survival rate for ESCC is still below 20%. Herein, ESCC‐specific DNA aptamers and an innovative aptamer‐modified nano‐system is introduced for targeted drug and gene delivery to effectively inhibit ESCC. The EA1 ssDNA aptamer, which binds robustly to ESCC cells with high specificity and affinity, is identified using cell‐based systematic evolution of ligands by exponential enrichment (cell‐SELEX). An EA1‐modified nano‐system is developed using a natural egg yolk lipid nanovector (EA1‐EYLNs‐PTX/siEFNA1) that concurrently loads paclitaxel (PTX) and a small interfering RNA of Ephrin A1 (EFNA1). This combination counters ESCC's proliferation, migration, invasion, and lung metastasis. Notably, EFNA1 is overexpressed in ESCC tumors with lung metastasis and has an inverse correlation with ESCC patient prognosis. The EA1‐EYLNs‐PTX/siEFNA1 nano‐system offers effective drug delivery and tumor targeting, resulting in significantly improved therapeutic efficacy against ESCC tumors. These insights suggest that aptamer‐modified nano‐systems can deliver drugs and genes with superior tumor‐targeting, potentially revolutionizing targeted therapy in ESCC.

## Graphical abstract

Ephrin A1 (EFNA1) is upregulated in esophageal squamous cell carcinoma (ESCC) with pulmonary metastasis and correlates with poor prognosis, meanwhile EA1 aptamer exhibits robust binding to ESCC cells with remarkable specificity. Consequently, they engineered an EA1‐modified nano‐system utilizing a natural egg yolk lipid nanovector (EA1‐EYLNs‐paclitaxel/siEFNA1) that offers enhanced drug/gene delivery and tumor targeting capabilities, exhibiting excellent ESCC cancer suppression efficiency.

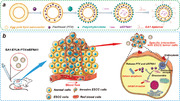

## 1 Introduction

Esophageal cancer ranks as the sixth most common cause of cancer‐related deaths. Notably, ≈ 95% of esophageal cancer cases in China are diagnosed as esophageal squamous cell carcinoma (ESCC).^[^ ^1^ ^]^ This subtype is marked by a high mortality rate and an unfavorable prognosis. The prevailing treatments for ESCC encompass surgery, chemotherapy, immunotherapy, and radiotherapy, as well as their combinations. However, these systemic treatments struggle to distinguish between normal and malignant cells, resulting in non‐specific cytotoxicity and subsequent side effects. The efficacy of these drugs is further diminished due to their inadequate targeting capability and inconsistent drug release within the ESCC tumor. As a consequence, the overall five‐year survival rate for ESCC remains dismally low, at less than 20%. This underlines the pressing need to develop more effective ESCC‐specific tools for targeted therapy to enhance esophageal cancer treatment outcomes.

The nano‐drug delivery system (NDDS), a prominent drug carrier, has garnered significant attention due to its unique benefits in cancer treatment. These benefits include increased drug solubility, enhanced drug distribution within tumor tissues, minimized cytotoxicity, the capacity to traverse biological barriers, and the potential for combined drug applications.^[^ ^2^ ^]^ Crucially, the efficacy and specificity of drug delivery can be amplified by attaching NDDS to tumor‐specific cell surface receptors. Examples of these receptors include folic acid and transferrin receptors,^[^ ^3^, ^4^ ^]^ RGD peptides,^[^ ^5^ ^]^ antibodies such as single‐chain antibody fragments and AMG 655 monoclonal antibodies,^[^ ^6^, ^7^ ^]^ glycans such as hyaluronic acid,^[^ ^8^ ^]^ and nucleic acid aptamers including EGFR and EpCAM aptamers.^[^ ^9^, ^10^ ^]^ These modified NDDSs target malignant cells while sparing healthy cells, thereby enhancing therapeutic efficacy. Building on our prior research,^[^ ^11^ ^]^ we introduced a natural egg yolk lipid nanovector (EYLNs). This vector exhibits high drug‐loading capacity, extended in vivo circulation, increased drug accumulation via the enhanced permeability and retention (EPR) effect, and improved intracellular penetration, all while maintaining high biocompatibility. It promises effective delivery of chemotherapy and nucleic acid drugs directly to tumor sites.

Metastasis stands as a pivotal reason for esophageal cancer treatment setbacks. In our previous research, we evidenced that Ephrin A1 (EFNA1) was more abundantly expressed in ESCC tissues from patients with lung metastasis than in those without. Furthermore, this overexpression inversely correlated with ESCC patient prognosis. Remarkably, when EFNA1 expression was suppressed, there was a significant decline in ESCC cell proliferation, migration, invasion, and lung metastasis both in vitro and in vivo. These observations suggest EFNA1 as a promising target for ESCC therapy. Given this understanding, we formulated EYLNs‐PTX/siEFNA1, encapsulating both the chemotherapy drug paclitaxel (PTX) and EFNA1 small interfering RNA (siEFNA1). In in vivo trials with tumor‐bearing mice, this compound showcased amplified therapeutic efficacy against ESCC, although its specific targeting capability for ESCC could be improved.

Aptamers, which can be single‐stranded DNA (ssDNA) or RNA oligonucleotides, are designed to selectively and robustly adhere to designated molecules or cells. The binding dynamics hinge on interactions such as hydrogen bonds, electrostatic forces, and van der Waals interactions.^[^ ^12^ ^]^ The systematic evolution of ligands by exponential enrichment (cell‐SELEX) protocol^[^ ^13^ ^]^ facilitates the derivation of aptamers with outstanding specificity and affinity by screening single‐stranded oligonucleotide libraries. Since Tuerk and Ellington's pioneering publication,^[^ ^14^ ^]^ the realm of aptamers has evolved into a burgeoning domain in targeted drug innovation. Aptamers offer distinct advantages, including (1) broad spectrum of target molecules, (2) remarkable affinity and specificity, (3) straightforward synthesis and modification, (4) minimal immunogenicity, and (5) efficient tumor penetration. Owing to these traits, aptamers are increasingly recognized as a leading approach for targeted nanoparticle drug delivery.^[^ ^15^, ^16^, ^17^ ^]^

**Scheme 1:**
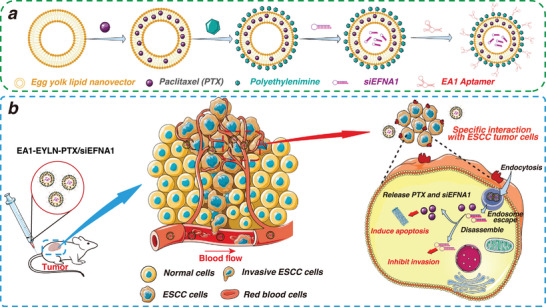
a) Preparation of aptamer EA1‐modified PTX/siEFNA1‐loaded nanomedicine. b) Schematic diagram of the proposed EA1‐EYLNs‐PTX/siEFNA1 by the modification of EYLNs with ESCC specific aptamer EA1 and co‐loading with PTX/siEFNA1 for enhanced therapeutic effect against ESCC.

In this study, our objective was to identify ESCC‐specific DNA aptamers and formulate a unique aptamer‐modified nano‐system to target drug and gene delivery, with the aim of inhibiting ESCC tumors. For the cell‐SELEX procedure, the poorly differentiated ESCC KYSE‐150 cell served as the positive selection, while the human esophageal epithelial cell (HEEC) was designated for negative selection. After seven selection cycles, five aptamers (EA1, EA2, EA3, EA4, and EA5) emerged. Flow cytometry analysis revealed that EA1 exhibited strong binding to various ESCC cell lines. Tissue immunofluorescence confirmed that EA1 could specifically detect clinical ESCC tissues, excluding adjacent tissues and other cancer types. This indicates that EA1 presents a potent targeting instrument for both ESCC diagnosis and targeted therapy. To leverage the targeting prowess of the aptamer, we crafted a novel EA1‐modified nano‐system (EA1‐EYLNs‐PTX/siEFNA1) by co‐loading PTX and siEFNA1 into a natural egg yolk lipid nanovector and then layering it with the EA1 aptamer (depicted in **Scheme** **1**). This nano‐system, EA1‐EYLNs‐PTX/siEFNA1, demonstrated efficient drug delivery combined with superior tumor‐targeting capabilities, leading to a notable boost in therapeutic efficacy against ESCC tumors both in vitro and in vivo. In sum, our results suggest that aptamer‐modified nano‐systems hold the promise of delivering drugs and genes with enhanced tumor targeting. This points a potential targeting tool in ESCC targeted therapy, opening avenues for promising transformations and applications.

## 2 Results and Discussion

### 2.1 EFNA1 is Overexpressed in ESCC with Pulmonary Metastasis and Correlates with Poor Patient Prognosis

**Figure 1:**
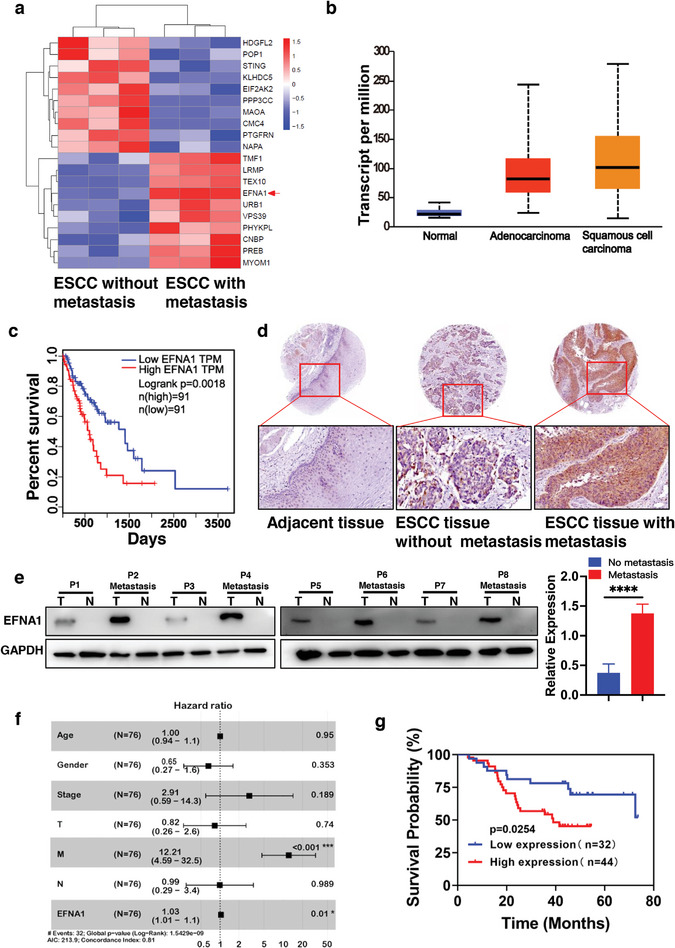
EFNA1 is overexpressed in esophageal squamous cell carcinoma with pulmonary metastasis and correlates with poor patient prognosis. a) Differentially expressed molecules in ESCC cancer and adjacent tissues using protein mass spectrographic analysis. b) EFNA1 mRNA expression in TCGA RNA‑seq database. c) Overall survival of patients displaying high or low/middle EFNA1 expression using GEPIA database. d) Expression of EFNA1 in ESCC cancer and adjacent normal tissues tested by IHC staining (magnification 200x). e) Expression levels of EFNA1 in ESCC tumor and normal tissues were determined using western blotting analysis. f) Forest plot of relationship between overall survival and different characteristics (Age, gender, TNM stage, and EFNA1 expression). g) Statistical analyses of association between EFNA1 expression and overall survival in 76 patients with ESCC. ^****^ *p* < 0.0001, compared with no metastasis group, were regarded as statistically acceptable.

To elucidate the potential molecular mechanism behind ESCC lung metastasis, we employed protein mass spectrometry to contrast the protein expressions in ESCC tissues with or without lung metastasis. The data indicated a pronounced overexpression of EFNA1 in ESCC tissues associated with lung metastasis (**Figure** **1a**). Corroborating this, an analysis of EFNA1 expression using The Cancer Genome Atlas (TCGA) database confirmed its elevated presence in both adenocarcinoma and squamous carcinoma esophageal tissues, as compared to standard tissues (Figure 1b). There was a marked negative correlation (*p* = 0.0018) between heightened EFNA1 expression in ESCC tissues and overall patient survival. Kaplan‑Meier survival analysis further revealed that patients exhibiting higher EFNA1 expression faces significantly diminished survival chances and unfavorable prognoses (Figure 1c).

Probing the clinical relevance of EFNA1 in ESCC, immunohistochemical staining was conducted on three paired samples of ESCC tissues and their neighboring tissues. The results displayed a considerably heightened expression of EFNA1 in ESCC tissues with lung metastasis relative to those without and the adjacent healthy tissues (Figure 1d). The expression levels of EFNA1 in ESCC tumor and normal tissues from 8 patients were also confirmed by western blotting (Figure 1e). In an additional step for validation, we monitored 76 primary ESCC patients over 5 years (with clinical details summarized in **Table** **1**). Forest plot (Figure 1f) of adjusted hazard ratios (aHRs) described that pathological stage (HR: 12.21, p<0.001) and EFNA1 expression (HR: 1.03, p = 0.01) are positively correlated with the overall survival. Employing a selected cut‐off value based on EFNA1 expression, we divided patients into two cohorts. The data revealed that the survival rate was discernibly lower (*p* = 0.0254) for those with heightened expression (Figure 1g). These findings underscore EFNA1's central role in ESCC lung metastasis, hinting at its potential as a diagnostic or prognostic marker for this condition.

**Table 1:** Relationship between EFNA1 expression and clinicopathological features of ESCC.

Clinical features	Cases (n)	EFNA1		*p* value
		Low	High	
All patients	76	32	44	
Sex				0.388
Male	58	26	32	
Female	18	6	12	
Age (years)				0.695
≤62	40	16	24	
>62	36	16	20	
Tumor location				0.941
Upper	5	2	3	
Middle	53	23	30	
Under	18	7	11	
Differentiation				0.392
G1	29	14	15	
G2/G3 G3	47	18	29	
TNM stage				0.082
I	3	3	0	
II	54	23	31	
III	19	6	13	

EFNA1 acts as the ligand for the tyrosine kinase receptor, EphA2.^[^ ^18^ ^]^ Contemporary research has also highlighted the interaction between EphA2 and EFNA1, which influences the progression of diverse tumors, including breast cancer^[^ ^19^ ^]^ and gastric tumors.^[^ ^20^ ^]^ Notably, over half of known proto‐oncogenes and oncogenes are tyrosine kinases. These play a pivotal role in modulating cell morphology, movement, and adhesion through cytoskeleton regulation.^[^ ^21^ ^]^ Such insights suggest EFNA1's significant involvement in tumor‐related neovascularization, invasion, and metastasis, positioning it as an appealing target in the realm of antitumor drug development.

### 2.2 EFNA1 Promotes ESCC Cell Proliferation, Migration, and Invasion

The protein expression of EFNA1 in HEEC and ESCC cell lines, namely KYSE‐30, KYSE‐150, KYSE‐410, and EC9706, was evaluated via Western blotting. The results revealed that EFNA1's expression in these ESCC cell lines was considerably elevated compared to HEEC cells (Figure S1a,b, Supporting Information), aligning with observations from clinical ESCC tissue analyses. To discern EFNA1's role in ESCC, KYSE‐150 cells underwent transient transfection with small interfering RNA (siRNA), leading to a marked reduction in EFNA1 protein expression (Figure S1c,d, Supporting Information). On further investigation, following EFNA1 knockdown, both the clonogenic capability (Figure S1e, Supporting Information) and proliferation rate (Figure S1f, Supporting Information) of KYSE‐150 cells decreased notably. This suggests EFNA1's stimulatory role in ESCC cell growth. Moreover, the migration and invasion capacities of si‐EFNA1 transfected KYSE‐150 cells, assessed through Transwell, were significantly reduced (Figure S1 g,h, Supporting Information) in comparison to si‐NC transfected cells. These findings uniformly suggest that inhibiting EFNA1 can deter the proliferation, stemness, migration, and invasion of ESCC cells.

**Figure 2:**
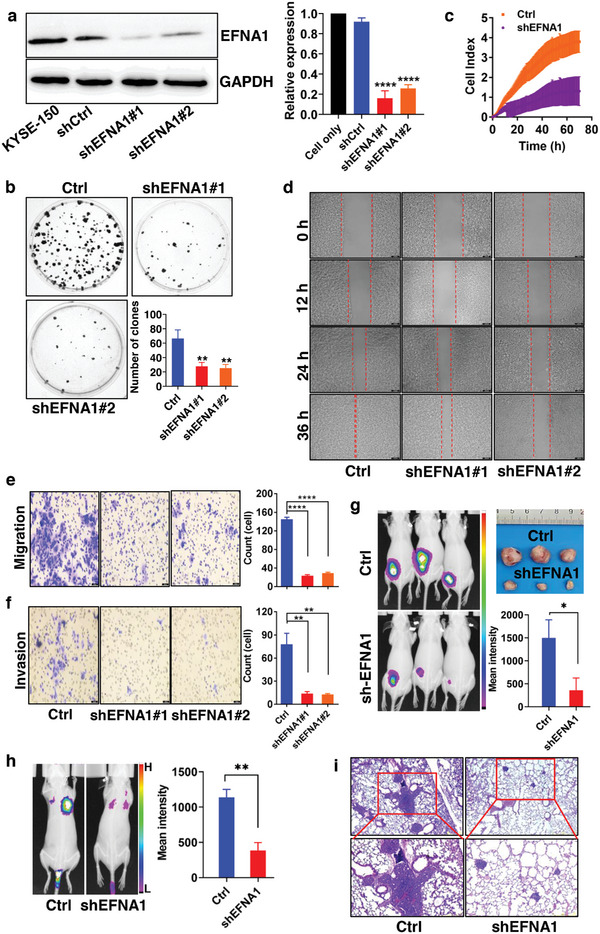
EFNA1 promotes ESCC cell proliferation, migration, and invasion. a) Relatively protein expression of EFNA1 by western blotting analysis in stable EFNA1‑knockdown KYSE‐150 cell line. b) Effect of EFNA1 on proliferation of shEFNA1‐transfected KYSE‐150 cells analyzed by colony‐formation assay. c) Proliferative ability of KYSE‐150 cells transfected with shEFNA1 evaluated using RTCA assay. d) Migration of shEFNA1‐transfected KYSE‐150 cells after transfection by wound‐healing assay. e, f) Effect of EFNA1 on migration and invasion of KYSE‐150 cells transfected with shEFNA1 tested by Transwell assays, penetrated cells were counted after 48 h of incubation and analyzed by Image J. g, h) Knockdown of EFNA1 inhibits the tumor growth, in vivo tumorigenicity of stable EFNA1‑knockdown KYSE‐150 cell line in xenograft subcutaneous tumor and lung metastases model were tested by live imaging. i) HE staining of lung metastases. ^*^ *p* < 0.05, ^**^ *p* < 0.01, ^****^ *p* < 0.0001, compared with shNC control group, were regarded as statistically acceptable.

Additionally, a KYSE‐150 cell line stably transfected with shEFNA1 was generated to further substantiate the influence of EFNA1 on ESCC cellular functions. Lentivirus plasmids employing the BR‑V‑108 vector (shEFNA1‐1 and shEFNA1‐2) were adeptly created to achieve stable EFNA1 silencing for subsequent evaluations. Silencing efficacy was validated through Western blotting (**Figure** **2**a). Upon EFNA1 suppression, both the clonogenic ability (Figure 2b) and proliferation (Figure 2c) of KYSE‐150 cells showed a substantial decline, reinforcing EFNA1's facilitative role in ESCC cell proliferation. Using wound‐healing assays (Figure 2d) and Transwell (Figure 2e and f), it was also observed that the migration and invasion of shEFNA1‐transfected KYSE‐150 cells markedly diminished. Cumulatively, these observations highlight EFNA1's role in promoting the proliferation, stemness, migration, and invasion of ESCC cells in vitro.

Of paramount importance, the inhibitory impact of EFNA1 silencing on tumor growth was assessed in KYSE‐150 tumor‐bearing mice. Monitoring subcutaneous tumors and lung metastases with an in vivo imaging system showed that both fluorescence intensity and tumor volume (Figure 2g,h) were significantly diminished compared to control mice. Hematoxylin and eosin (HE) staining (Figure 2i) also displayed reduced lung metastasis in the shEFNA1‐transfected KYSE‐150 cell group. In summary, these results underscore that downregulating EFNA1 substantially curtails both the formation of ESCC subcutaneous tumors and their metastatic potential to the lungs, mirroring the in vitro findings. These observations reaffirm EFNA1's instrumental role in ESCC progression, aligning with prior research concerning EFNA1's involvement in other tumors.^[^ ^19^, ^20^ ^]^ Thus, EFNA1 emerges as a potential therapeutic molecular target.

### 2.3 ESCC‐specific Aptamer Screening, Affinity, Specificity, and Tissue Targeting Analysis

Aptamers are recognized for their high binding affinity, specificity, and minimal immunogenicity, making them promising targeted ligands for drug delivery. Additionally, aptamers not only enhance nanomedicine distribution in targeted tissues but also improve drug penetration. For instance, the EGFR aptamer has been employed for drug targeting in conditions such as pancreatic ductal adenocarcinoma^[^ ^22^ ^]^ and triple‐negative breast cancer cells.^[^ ^23^ ^]^ Similarly, HER2 aptamer‐mediated nanomedicine targets HER2‐positive breast cancer,^[^ ^24^ ^]^ and PSMA‐mediated nanomedicine addresses prostate cancer.^[^ ^25^ ^]^ In a previous study, our group developed an EGFR‐modified RNA nanomedicine for ESCC treatment.^[^ ^26^ ^]^ However, the EGFR aptamer, while effective, lacks specificity for ESCC.

#### 2.3.1 Cell‐SELEX Process

**Figure 3:**
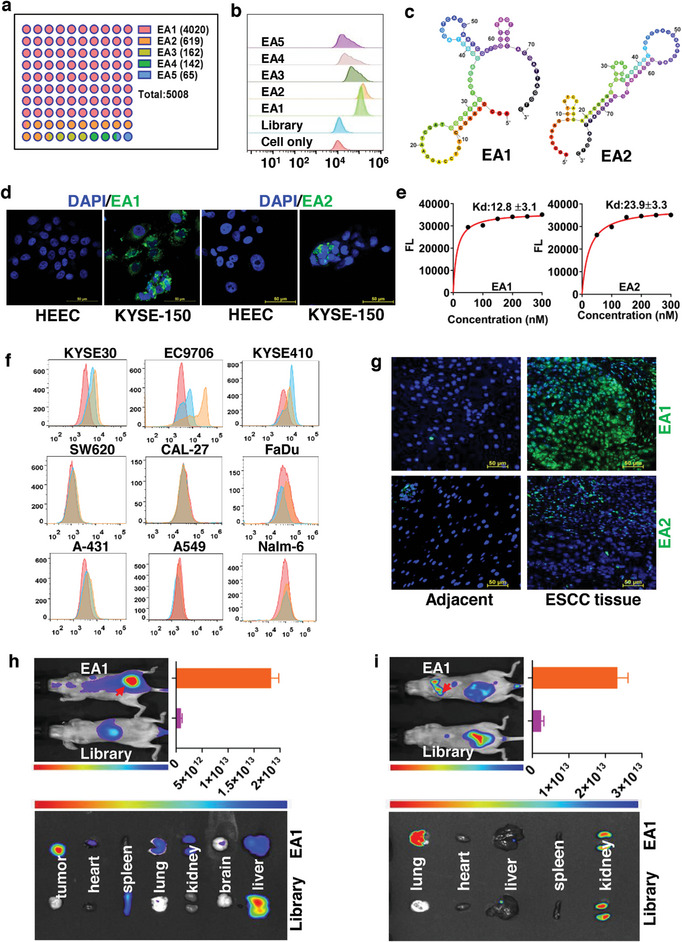
ESCC‐specific aptamer screening, affinity, specificity, and tissue targeting analysis. a) High enrichment of selection library, selected aptamers were named EA1, EA2, EA3, EA4, and EA5. b) Flow cytometry assays of FAM‐labeled aptamer candidates to target KYSE‐150 cells. c) Secondary structures of EA1 and EA2 predicted by RNAfold. d) LSCM imaging of target KYSE‐150 and control HEEC cells incubated with 250 nM FAM‐labeled EA1 and EA2. e) Determination of dissociation constant (K_d_) of EA1 and EA2 to KYSE‐150 cells using flow cytometry. f) Binding assays of FAM‐labeled aptamer EA1 (blue line) and EA2 (yellow line) to different cell lines by flow cytometry. FAM‐labeled random ssDNA (red line) was used as control. g) Confocal imaging of paired ESCC clinical tissues with FAM‐labeled EA1 and EA2 compared with paired adjacent tissues. h) In vivo targeting ability and biodistribution of the aptamer EA1 in xenograft subcutaneous tumor model. i) In vivo targeting ability and biodistribution of aptamer EA1 in xenograft lung metastases model.

The cell‐SELEX process, (depicted in Figure S2a, Supporting Information), was executed using established methods, with minor alterations, to identify aptamers with specific binding affinity for ESCC cells.^[^ ^27^ ^]^ The poorly differentiated ESCC KYSE‐150 cell served as the target for positive selection, while the HEEC was the control for negative selection. The evolution of the selected ssDNA library was monitored using flow cytometry. After 15 selection rounds, the ssDNA pool underwent amplification, sub‐cloning and sequencing. Enrichment results from the selection library (**Figure** **3**a) revealed a total of 5008 sequenced ssDNA sequences. The top five sequences, based on copy number and family distribution EA1, EA2, EA3, EA4, and EA5 (detailed in **Table** **2**) were shortlisted as potential aptamer candidates for binding analysis with KYSE‐150 cells. The fluorescence signals from EA1, EA2, EA3, EA4, and EA5 displayed markedly stronger binding to KYSE‐150 cells compared to random library sequences (Figure 3b), with negligible fluorescence interaction with HEEC cells (Figure S2b, Supporting Information). This suggests that the selected candidates exhibit significant binding affinities to KYSE‐150 cells rather than HEEC cells. The aptamer secondary structures for EA1, EA2, EA3, EA4, and EA5 were predicted using mFold software (Figure 3c; Figure S2c, Supporting Information). Analysis revealed that EA1 and EA2 showcased superior binding efficiency and enrichment, leading to their selection for further characterizations.

**Table 2:** Sequences of aptamer candidates.

Name	Random‐region sequence (5′‐3′)
EA1	5′‐AGCCTAAGCCTGTCCAGGAATCGATGGCTTAGTGGCACGATTAGGTCAGGAATCGATGGCTTAGTGGCACGATTAGGTC‐3′
EA2	5′‐AGCCTAAGCCTGTCCAGGAATCGTGCAGCCATAGCCTAAGCCTGTCCAGGAATCGATGGCTTAGTGGCACGATTAGGTC‐3′
EA3	5′‐AGCCTAAGCCTGTCCAGGAATCGATGGCTTAGCCTAAGCCTGTCCAGGAATCGGCATGGCTTAGTGGCACGATTAGGTC‐3′
EA4	5′‐AGCCTAAGCCTGTCCAGGAATCGATGGCTTAGCCTAAGCCTGTCCAGGAATCGCCATGGCTTAGTGGCACGATTAGGTC‐3′
EA5	5′‐AGCCTAAGCCTGTCCAGGAATCGGTGCGCACTAGCCTAAGCCTGTCCAGGAATCGATGGCTTAGTGGCACGATTAGGTC‐3′

#### 2.3.2 Binding Affinity

To determine the optimal binding conditions, the incubation times and temperatures of the chosen aptamers with KYSE‐150 cells were explored (data not presented). The findings indicated an ideal incubation time of 60 min at 4 °C for subsequent evaluations. Notably, a robust binding capability was still evident at 37 °C, suggesting potential for in vivo usage. Confocal images (Figure 3d) highlighted the specific binding capacities of EA1 and EA2 to cells, as evidenced by the prominent green fluorescence signals detected on the target KYSE‐150 cells but absent in the control HEEC cells. Further, to quantitatively gauge the binding affinities of the aptamers, the dissociation constant (K_d_) values were established (Figure 3e; Figure S2d, Supporting Information). Values in the nanomolar range (EA1, 12.8 ± 3.1 nM; EA2, 23.9 ± 3.3 nM; EA3, 32.08 ± 5.5 nM; EA4, 45.9 ± 6.0 nM; and EA5, 73.9 ± 26.3 nM) were recorded, signifying the superior binding capacities of the chosen aptamers.

#### 2.3.3 Binding Selectivity

To assess the binding specificity of the identified aptamers, various ESCC cells, other cancer cells, and leukocytes from a healthy volunteer were treated with the FAM‐labeled aptamers for recognition tests. Flow cytometry outcomes revealed that EA1 and EA2 primarily bonded with specific ESCC cell lines (KYSE‐30, KYSE‐410, and ECa9706) but exhibited minimal affinity to other cancer cell lines, including SW620, CAL‐27, FaDu, A‐431, A549, and Nalm‐6 (Figure 3f). Additionally, minimal binding to lymphocytes, monocytes, and neutrophils was noted (Figure S2e, Supporting Information), which suggests that EA1 and EA2 exhibit commendable stability and biocompatibility in vivo. Furthermore, EA1 demonstrated a more robust binding capability compared to EA2 with ESCC cells. Overall, aptamer EA1 emerged as a promising and specific detection tool for ESCC cells.

#### 2.3.4 Clinical ESCC Tissues Imaging

Inspired by the potent and specific binding of EA1 and EA2 to ESCC cells, multiple ESCC tissues along with corresponding adjacent tissues were acquired to evaluate the potential of aptamers in clinical tissue pathological assessments. All tissue sections underwent HE staining and were validated by a pathologist prior to examination. These sections, paired with adjacent tissues, were then treated with FAM‐labeled EA1 and EA2 for confocal imaging. The ESCC tissue samples exhibited prominent green fluorescence signals after treatment with FAM‐labeled aptamers, in contrast to the adjacent tissues which showed subdued fluorescence (Figure 3g). Notably, EA1 exhibited superior binding to ESCC tissues, highlighting its potential for ESCC tissue detection. To further ascertain EA1's specificity, various tumor tissue sections, including ESCC and other types like gastric, liver, colon, and lung cancers, along with their adjacent tissues, were subjected to Cy5.5‐labeled EA1 for confocal imaging. The obtained images (Figure S2f, Supporting Information) confirmed pronounced signal disparities between ESCC tissues and their counterparts, with negligible fluorescence signals in other tumor types and paired adjacent tissues. This underscores EA1's potential as a diagnostic probe specifically tailored for ESCC tissue imaging.

#### 2.3.5 In vivo Targeting Ability

The inherent ability of aptamers to target in vivo is paramount. While Chen et al. identified ssDNA aptamers for ESCC targeting, comprehensive in vivo tissue targeting evaluations remain pending. Furthermore, the targeted drug delivery dynamics of the said nanomedicine have not been detailed.^[^ ^28^ ^]^ In this study, both xenograft subcutaneous and lung metastasis mouse models were employed. Subsequently, 400 nmol of Cy5.5‐labeled EA1 was administered intravenously into ESCC mouse models to affirm the in vivo targeting prowess of the selected aptamer. Predictably, Cy5.5‐labeled EA1 consistently converged at the tumor sites. This suggests that EA1 could efficiently localize in both the subcutaneous tumor tissues (Figure 3h) and lung metastasis tissues (Figure 3i) associated with ESCC. Such findings might be attributed to EA1's high affinity for its target cells, potentially safeguarding the probes from metabolic breakdown.^[^ ^29^ ^]^ Collectively, EA1 holds promise as an ESCC‐specific diagnostic tool and as a molecule adept at in vivo ESCC tumor targeting.

### 2.4 Biodistribution of EA1‐Mediated Nanoparticle

**Figure 4:**
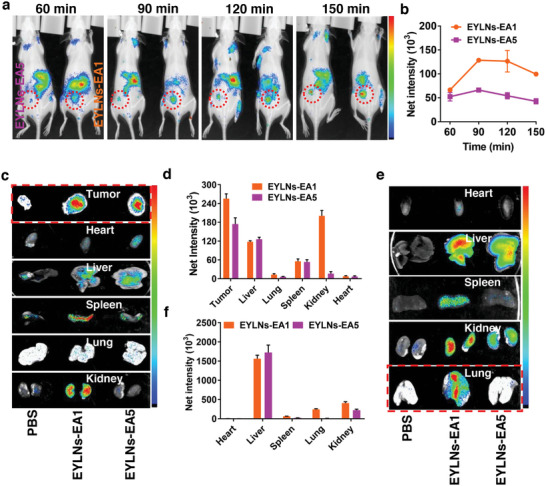
In vivo biodistribution of aptamer‐modified EYLNs. a) In vivo time‐dependent fluorescence imaging in KYSE‐150‐derived tumor xenograft models. KYSE‐150 tumor‐bearing mice intravenously injected with EYLNs‐Cy5.5‐EA1 and EYLNs‐Cy5.5‐EA5 (control), then fluorescence images of the dorsal side were captured and quantified at 60, 90, 120, and 150 min by live imaging system. b) Pharmacokinetic profiles of EYLNs‐Cy5.5‐EA1 or EYLNs‐Cy5.5‐EA5 at tumor sites calculated by using fluorescence intensities. c, d) Biodistribution and quantification of EYLNs‐Cy5.5‐EA1 and EYLNs‐Cy5.5‐EA5 in KYSE‐150 subcutaneous tumor‐bearing mice. e, f) Biodistribution and quantification of EYLNs‐Cy5.5‐EA1 and EYLNs‐Cy5.5‐EA5 in KYSE‐150‐lung metastasis mice. Fluorescence images of tumor and heart, liver, spleen, lungs, and kidneys were captured and quantified.

In our prior study,^[^ ^11^ ^]^ we introduced a natural EYLN that exhibited extended in vivo circulation and heightened tumor accumulation via the EPR effect, making it an ideal vehicle for delivering chemotherapy drugs and genes to tumor sites. In this study, we sought to determine the potential of EA1 in directing EYLNs toward ESCC tumors. To begin, Cy5.5‐EA1 and Cy5.5‐EA5 (as a control aptamer) modified EYLNs were synthesized. Subsequently, xenograft subcutaneous and lung metastasis mouse models were established. The mice were then intravenously administered with EYLNs‐Cy5.5‐EA1 and EYLNs‐Cy5.5‐EA5. Both EA1 and EA5 were capable of directing EYLN delivery to tumor‐laden tissues. The fluorescence intensity at the tumor site was notably higher for the EYLNs‐Cy5.5‐EA1 compared to the EYLNs‐Cy5.5‐EA5 control group (**Figure** **4**a,b). A comparative evaluation between EYLNs‐Cy5.5‐EA1 and EYLNs‐Cy5.5‐EA5 revealed marked disparities in organ and tumor fluorescence intensities (Figure 4c). Quantitative metrics indicated a superior tumor accumulation of EYLNs‐Cy5.5‐EA1 over EYLNs‐Cy5.5‐EA5 (Figure 4d). Additionally, EYLNs‐Cy5.5‐EA1 demonstrated a commendable ability to target lung metastases in the lung metastasis mouse model, a feat not achieved by EYLNs‐Cy5.5‐EA5 (Figure 4e,f). These findings reinforce the notion that introducing EA1 could significantly boost the targeted delivery and accumulation of EYLNs at ESCC tumors, optimizing nanomedicine delivery in vivo.

### 2.5 Construction and Characterization of EA1‐Modified PTX/siEFNA1‐Loaded Nanomedicine

**Figure 5:**
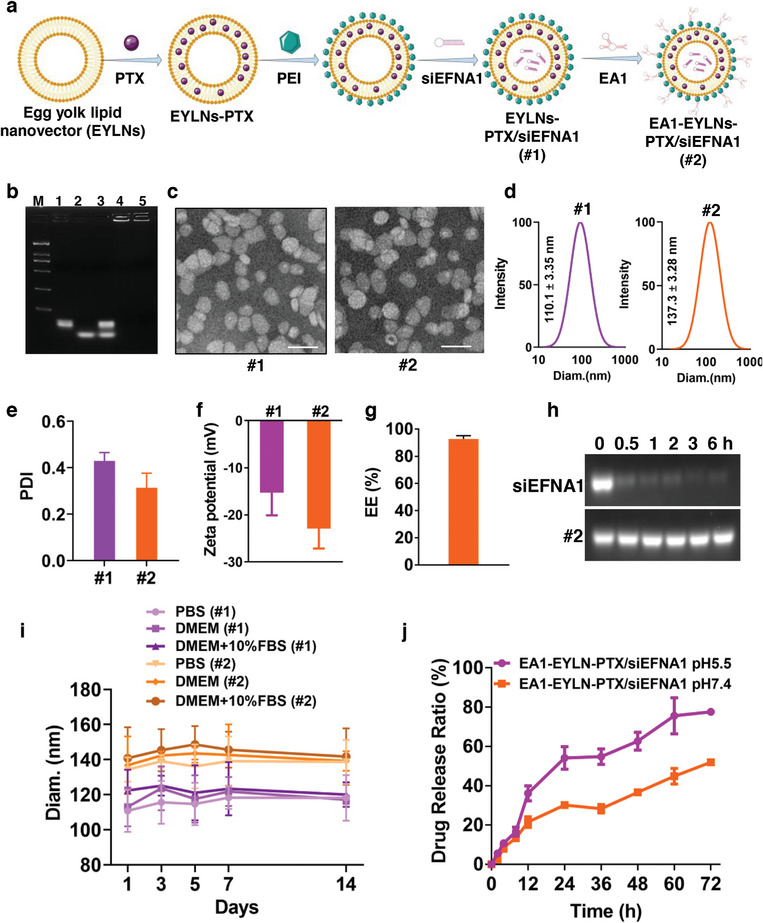
Preparation and physicochemical characterization of EA1‐modified PTX/siEFNA1‐loaded nanomedicine. a) Schematic illustration of EA1‐EYLNs‐PTX/siEFNA1 through lipid film dispersion‐hydration method. b) 3% Agarose electrophoresis to identify conjugation of aptamer EA1 and siEFNA1 to EYLNs. M: DNA ladder, 1: Free EA1, 2: Free siEFNA1, 3: Simple mixture of EYNs, EA1 and siEFNA1, 4: EA1‐EYLNs‐PTX/siEFNA1, 5: EA1‐EYLNs‐siEFNA1. c) Morphology observations (TEM images, Scale bar: 100 nm) of EYLNs‐PTX/siEFNA1 (#1) and EA1‐EYLNs‐PTX/siEFNA1 (#2). d) Particle size distribution of EYLNs‐PTX/siEFNA1 and EA1‐EYLNs‐PTX/siEFNA1 using DLS analysis. e) Polydispersity index (PDI) of EYLNs‐PTX/siEFNA1 and EA1‐EYLNs‐PTX/siEFNA1. f) Surface ζ potentials of EYLNs‐PTX/siEFNA1 and EA1‐EYLNs‐PTX/siEFNA1. g) Encapsulation efficiency of PTX by EA1‐EYLNs‐PTX/siEFNA1. h) Enzymatic stability of EA1‐EYLNs‐PTX/siEFNA1 from RNase A degradation. i) Stability of EYLNs‐PTX/siEFNA1 and EA1‐EYLNs‐PTX/siEFNA1 tested by measuring particle size in PBS, DMEM, and DMEM with 10% FBS at 37 °C. j) In vitro drug release profiles of PTX from EA1‐EYLNs‐PTX/siEFNA1 in different PBS buffer solution (pH 5.5 and pH 7.4).

From the results outlined above, it is evident that using EA1‐mediated molecular therapy to target EFNA1, in tandem with chemotherapy, might more effectively curb the progression of ESCC. Drawing inspiration from our earlier work on aptamer‐modified nanovectors targeting HER2^+^ breast cancer,^[^ ^30^ ^]^ we developed a liposomal nanovector designed with a surface‐conjugated targeting ssDNA aptamer. This nanovector is engineered for the precise co‐delivery of first‐line chemotherapeutics PTX and siEFNA1 into ESCC cells. The EA1‐modified liposomal complexes were synthesized using a lipid film dispersion‐hydration method, with the procedure outlined in a schematic illustration (**Figure** **5**a).

Based on our earlier research,^[^ ^11^ ^]^ we delved into formulating the EA1‐modified PTX/siEFNA1‐loaded EYLNs to fine‐tune the ratios of PTX, PEI, and EA1. Initially, visual assessments indicating that escalating PTX amounts (ranging from 60 µg to 600 µg) led to layer separation, potentially due to the encapsulation constraints of EYLNs. However, observations over a span of 48 h showed that the 60 µg and 120 µg batches remained stable, without any sedimentation or layer separation (Figure S3a, Supporting Information). This stability was further affirmed by the particle size increase (Figure S3b, Supporting Information). To strike a balance between optimal PTX loading and stability, the 120 µg PTX was earmarked for further fine‐tuning with siRNA and aptamer. Second, for efficient siRNA and aptamer loading onto EYLNs, a cationic procedure was introduced. Mixing 3 mg EYLNs with varying PEI amounts formed a cationic liposome, followed by the addition of 1.8 nmol siEFNA1 and 4.5 nmol EA1. As the PEI amounts (spanning 20 µg to 288 µg) increased, there was a notable decline in the brightness of the unloaded siRNA bands, evident at the base of the 2% agarose gel (Figure S3c, Supporting Information). This suggests that at a PEI mass of 144 µg, siEFNA1 were efficiently loaded into EYLNs. Last, to gauge the ideal EA1 mass for modification, varying amounts were tested. It was observed that within a range of 3.5 nmol to 4.5 nmol, EA1 aptly modified the EYLNs. However, going beyond this range compromised the aptamer modification, a finding supported by the unloaded siRNA observed at the gel's base in lanes 7, 8, 9 and 10 (Figure S3d, Supporting Information). In summary, the optimal mass ratios for EYLNs/PEI/siEFNA1, modified EA1, and the loaded PTX were determined to be 3 mg EYLNs: 144 µg PEI: 1.8 nmol siEFNA1, 4.5 nmol EA1, and 120 µg PTX, respectively. Following these optimized parameters, EYLNs‐PTX/siEFNA1 (#1) and EA1‐EYLNs‐PTX/siEFNA1 (#2) were synthesized and then purified of free PTX and unbound EA1 aptamers through GelFiltration Chromatography. Figure 5b illustrates that the free EA1 aptamer and siEFNA1 each presented a distinct band. Meanwhile, EYLNs‐PTX/siEFNA1 and EA1‐EYLNs‐PTX/siEFNA1 remained predominantly at the starting point, validating the efficient conjugation of siEFNA1 and EA1 to EYLNs.

Subsequent tests were conducted to determine the physicochemical properties of EYLNs‐PTX/siEFNA1 (#1) and EA1‐EYLNs‐PTX/siEFNA1 (#2). This involved analyzing their morphology, size distribution, ζ potential, and encapsulation efficacy. Transmission‐electron microscope (TEM) imagery (Figure 5c) showed that both complexes were of consistent size, spherical in shape, and had a singular structure. Corroborating these visual findings, the mean particle sizes for EYLNs‐PTX/siEFNA1 and EA1‐EYLNs‐PTX/siEFNA1 stood at 110.1 ± 3.35 nm and 137.3 ± 3.28 nm, with a satisfactory particle size distribution, polydispersity index (PDI) (as seen in Figure 5d,e). Figure S3e (Supporting Information) showcases the particle size distributions of other carriers. The observed enlargement in particle size for EA1‐EYLNs‐PTX/siEFNA1 aligns with past studies,^[^ ^31^ ^]^ suggesting that siRNA's conjugation to liposomes through PEI cationization enlarges the nanocarrier's size.

One critical metric for appraising the properties of nanomedicine is stability. The ζ potential serves as a key gauge for this, among other factors.^[^ ^32^ ^]^ Ideally, for nanomedicine to be deemed stable, the absolute ζ potential should exceed 15, symbolizing resistance to clustering due to the electrostatic repulsion between like‐charged dispersed particles. The measured ζ potential for EA1‐EYLNs‐PTX/siEFNA1 was −22.92 ± 4.23 mV, indicating commendable dispersion stability. Furthermore, the ζ potential for EA1‐EYLNs‐PTX/siEFNA1 was considerably lower than that for EYLNs‐PTX/siEFNA1 (−22.92 ± 4.23 mV versus −15.27 ± 4.83 mV, as portrayed in Figure 5f). This signifies that negatively charged ssDNA aptamers were successfully attached to the surface of EA1‐EYLNs‐PTX/siEFNA1. Post quantification, the encapsulation efficiency for PTX within EA1‐EYLNs‐PTX/siEFNA1 was pegged at 92.7 ± 2.41%, as denoted in Figure 5g. To assess the nuclease resistance of EA1‐EYLNs‐PTX/siEFNA1 in vivo, DMEM media containing 10% fetal bovine serum and RNase buffer were used to simulate blood stability. In the presence of RNase, free siEFNA1 showed decreasing band brightness over time (Figure 5h), signifying its degradation. In contrast, minimal degradation was observed with EA1‐EYLNs‐PTX/siEFNA1, highlighting the RNase A resistance of EYLNs. This underscores that nucleic acids (either siRNA or ssDNA) attached to liposomes possess improved resilience against nuclease degradation. During stability analysis of EA1‐EYLNs‐PTX/siEFNA1 and EYLNs‐PTX/siEFNA1 in PBS, DMEM media, and DMEM with 10% fetal bovine serum at 37 °C, only minor particle alterations were noted (Figure 5i).

The in vitro release patterns of PTX from EA1‐EYLNs‐PTX/siEFNA1 were examined under varying pH levels (Figure 5j). Under physiological conditions (pH 7.4), the release of PTX was consistently gradual with extended incubation. However, in an acidic setting (pH 5.5), mimicking the tumor microenvironment (TME), there was a pronounced PTX release between 12 to 72 h. This indicates that EA1‐EYLNs‐PTX/siEFNA1 retains its structure and exhibits steady drug release at neutral pH, but displays marked release when the pH drops from 7.4 to 5.5. Such behavior is advantageous for aptamer‐modified liposome delivering in lower pH conditions.^[^ ^17^ ^]^ Given the slightly acidic nature of tumor environments, scientists have designed acid‐responsive nanomedicines^[^ ^33^, ^34^ ^]^ to enhance drug release specifically in tumors. Our preliminary experiments showed that EYLNs are non‐covalently linked to Doxorubicin's hydroxyl group due to its surface carboxyl group. This bond weakens in acidic conditions, promoting higher drug release.^[^ ^35^ ^]^ Testing EA1‐EYLNs‐PTX/siEFNA1 in PBS at diverse pH revealed increased PTX release in acidic settings, likely due to this mechanism.

### 2.6 Intracellular Uptake, Endocytosis, Endosomal Escape, and Tumor Penetration of EA1‐modified PTX/siEFNA1‐loaded Nanomedicine

#### 2.6.1 Intracellular Uptake

**Figure 6:**
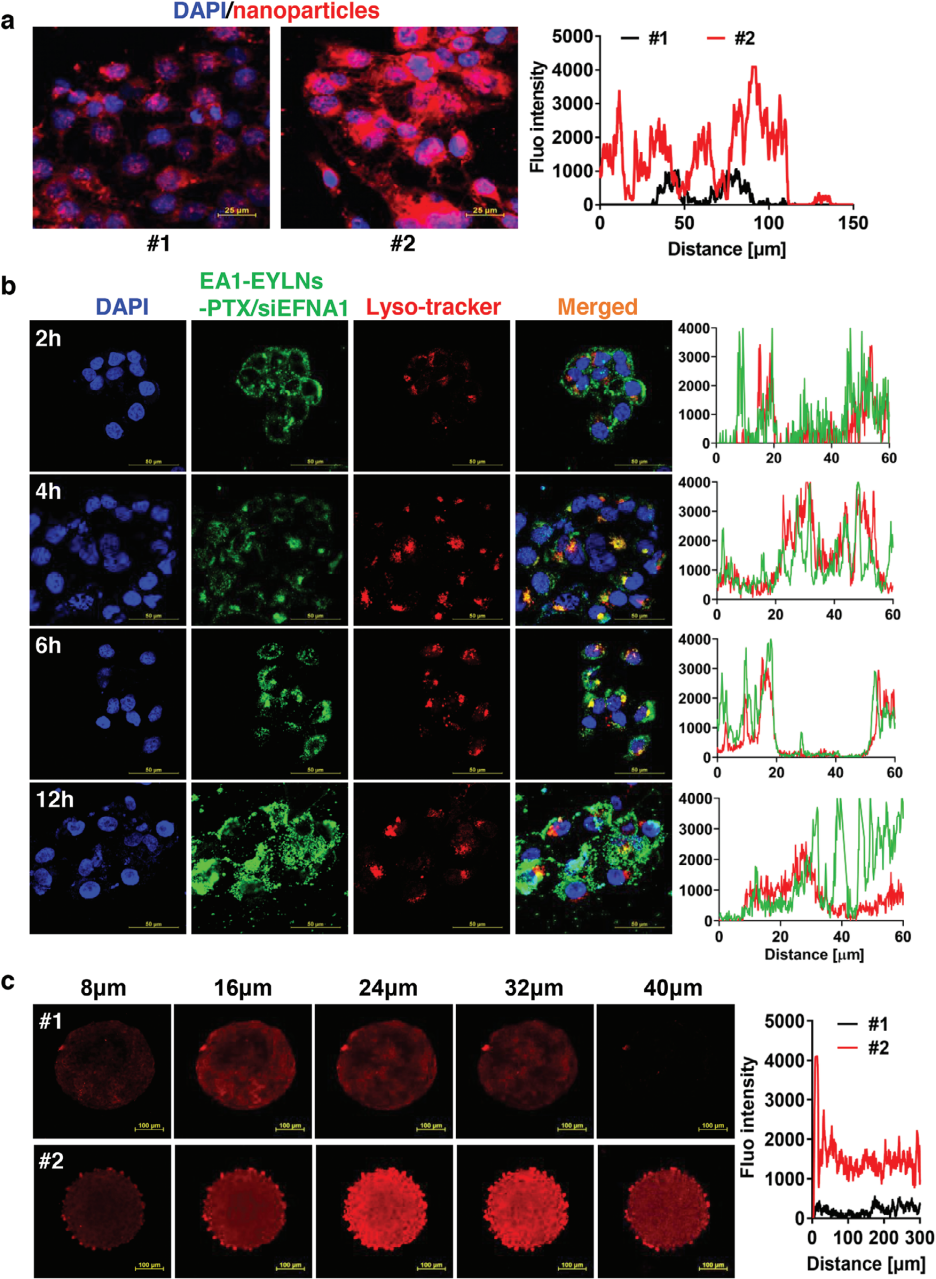
Intracellular uptake, endocytosis, endosomal escape, and tumor penetration of EA1‐modified PTX/siEFNA1‐loaded nanomedicine. a) Intracellular uptake of PKH26 labeled EYLNs‐PTX/siEFNA1 (#1) and EA1‐EYLNs‐PTX/siEFNA1 (#2) in KYSE‐150 cells using confocal imaging. Scale bar: 25 µm. b) Endocytosis and endosomal escape of EA1‐EYLNs‐PTX/siEFNA1 by observing co‐localization of PKH26 labeled EYLNs and LysoTracker labeled lysosomes in KYSE‐150 cells. Cells were incubated with EA1‐EYLNs‐PTX/siEFNA1 for 2 h, 4 h, 6 h, and 12 h at 37 °C, and lysosomes were subsequently stained with LysoTracker Red. Nuclei were stained with DAPI. Scale bar: 50 µm. c) Tumor penetration ability of PKH26 labeled EYLNs‐PTX/siEFNA1 (#1) and EA1‐EYLNs‐PTX/siEFNA1 (#2) in KYSE‐150 cell spheroids. KH26 labeled EYLNs‐PTX/siEFNA1 or EA1‐EYLNs‐PTX/siEFNA1 were incubated with KYSE‐150 cells or spheroids for 12 h at 37 °C, respectively. EYLNs distribution was monitored using red fluorescence,  and immunofluorescence intensities of PKH26 were analyzed. Scale bar: 100 µm.

Effective tumoricidal effects require nanomedicine to bind to the target cells and enter them. The drug distribution of EA1‐modified PTX/siEFNA1‐loaded nanomedicine in KYSE‐150 cells was examined to determine if EA1 modification enhanced binding and uptake. After incubating PKH26 labeled EYLNs‐PTX/siEFNA1 and EA1‐EYLNs‐PTX/siEFNA1 with KYSE‐150 cells for 6 h at 37 °C, confocal images were obtained using laser scanning confocal microscope (LSCM). Interestingly, EA1‐EYLNs‐PTX/siEFNA1 treated cells displayed prominent red fluorescence, more than the EYLNs‐PTX/siEFNA1 treated cells (**Figure** **6**a). This suggests enhanced intracellular accumulation of the EA1‐modified nanovector compared to EYLNs‐PTX/siEFNA1. Additionally, while EYLNs‐PTX/siEFNA1 mainly settled in the periphery and cytoplasm of KYSE‐150 cells, EA1‐EYLNs‐PTX/siEFNA1 notably accumulated in the nucleus within the 6 h incubation. These results suggest that EA1 modification improves EYLNs distribution and enables targeted delivery of PTX and siEFNA1 to KYSE‐150 cancer cells.

#### 2.6.2 Endocytosis and Endosomal Escape

Post intracellular uptake, transporting siEFNA1 and PTX from endo/lysosomes to the cytoplasm becomes pivotal for gene silencing and cancer suppression. The endo/lysosomal escape of these payloads in KYSE‐150 cells was observed over varying incubation times (2 h, 4 h, 6 h, and 12 h) using LSCM. PKH67 labeled EA1‐EYLNs‐PTX/siEFNA1 are represented in green, while LysoTracker‐stained endo/lysosomes appear red. As depicted in Figure 6b, after 2 h of incubation, most EA1‐EYLNs‐PTX/siEFNA1 (labeled by PKH67 Green) remained on cell membranes. At the 4 h mark, increased cytoplasmic presence of EA1‐EYLNs‐PTX/siEFNA1 was noted, with a strong orange hue emerging from the fluorescence overlap of LysoTracker Red and PKH67 Green. This suggests that EA1‐EYLNs‐PTX/siEFNA1 enters via the endolysosomal pathway, with most NDDS unable to escape from endo/lysosomes. However, by 12 h, this overlap reduced, evidenced by diminished orange fluorescence, indicating separation of red (endo/lysosome) and green (EA1‐EYLNs‐PTX/siEFNA1) hues. This implies that EA1‐EYLNs‐PTX/siEFNA1 can break away from endo/lysosomal membranes, facilitating siEFNA1 and PTX accumulation in the cytoplasm.

#### 2.6.3 Tumor Penetration

The dense stroma of the ESCC extracellular matrix hinders the penetration of chemotherapy drugs. Even though most nanotherapeutics can passively accumulate at tumor sites, they predominantly concentrate near the peripheral tumor blood vessels, limiting deep tumor penetration.^[^ ^36^ ^]^ While various strategies exist to increase tumor permeability, including nanomedicine design and tumor microenvironment modulation,^[^ ^37^ ^]^ our previous findings highlight that the EA1 aptamer has robust tissue permeability. This indicates that aptamer modification holds great promise as a strategy for enhancing tumor penetration. To replicate the pathological penetration barrier in ESCC, we constructed 3D‐tumor spheroids of KYSE‐150 cells in vitro. We then treated the KYSE‐150‐3D tumor spheroid with Cy5‐labeled EYLNs for 12 h to gauge penetration ability. LSCM Z‐stack scanning, shown in Figure 6c, revealed that EYLNs‐PTX/siEFNA1 primarily situated itself on the spheroid's exterior, with sparse distributions within. Conversely, EA1 notably enhanced EYLN‐PTX/siEFNA1 distribution within deep ESCC spheroids, likely owing to the cell‐penetrating attribute of the EA1 aptamer. This implies that our EA1‐EYLN‐PTX/siEFNA1 offers improved in vivo solid tumor penetration.

### 2.7 In Vitro ESCC Cancer Suppression Efficiency

**Figure 7:**
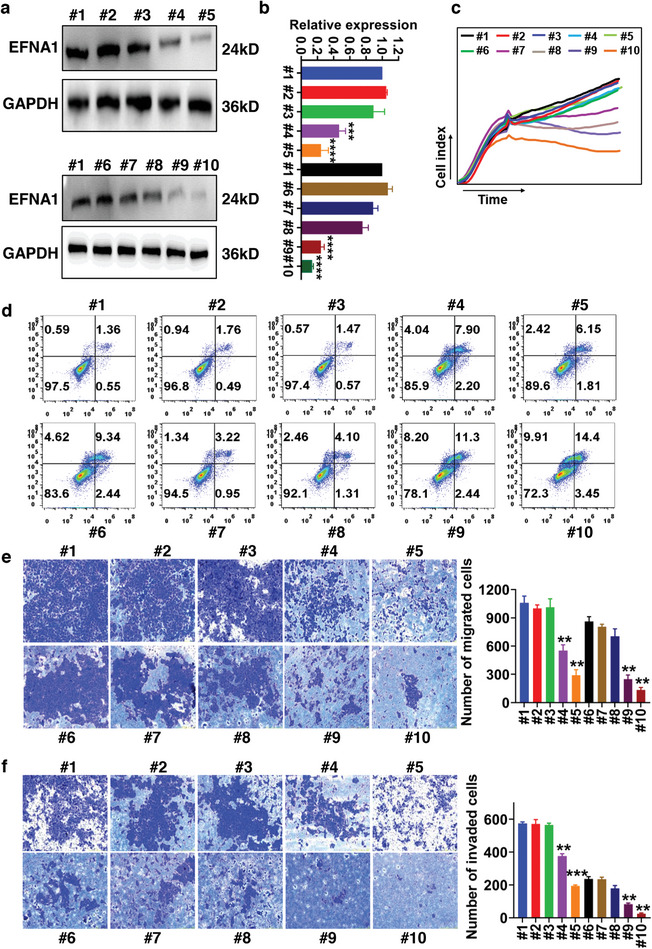
In vitro ESCC cancer suppression efficiency of EA1‐modified PTX/siEFNA1‐loaded nanomedicine. a, b) Expression of EFNA1 in KYSE‐150 cells after treating with various NDDS. c) Proliferative ability of KYSE‐150 cells after treating with various NDDS evaluated using RTCA assay. d) The representative scattering plots of apoptotic assay of KYSE‐150 cells treated with free drugs or various NDDS using Annexin V‐FITC apoptosis kit. e, f) Inhibition effects of various NDDS on migration and invasion of KYSE‐150 cells tested by Transwell assays, penetrated cells were counted after 48 h of incubation and analyzed by Image J. Treatments includes PBS (#1), EYLNs (#2), EA1‐EYLNs (#3), EYLNs‐siEFNA1 (#4), EA1‐EYLNs‐siEFNA1 (#5), PTX (#6), EYLNs‐PTX (#7), EA1‐EYLNs‐PTX (#8), EYLNs‐PTX/siEFNA1 (#9), and EA1‐EYLNs‐PTX/siEFNA1 (#10). ^**^ *p* < 0.01, ^***^ *p* < 0.001,^****^ *p* < 0.0001, compared with PBS group, were regarded as statistically acceptable.

The suppressive effectiveness of EA1‐modified PTX/siEFNA1‐loaded nanomedicine was assessed using Western blotting, real‐time cell analysis (RTCA), flow cytometry, and Transwell assays on KYSE‐150 cells in vitro. The efficient delivery of siEFNA1, augmented by EA1 modification, markedly reduced EFNA1 expression in ESCC cells (**Figure** **7**a,b). Moreover, exposure of KYSE‐150 cells to various nanomedicines and PTX showed that siEFNA1 delivery boosted PTX's capability to curtail KYSE‐150 cell proliferation, migration, invasion and enhance the apoptosis level. The effect was even more pronounced with EA1 modification (Figure 7c–f). This suggests that EA1 can enhance nanomedicine binding efficiency, leading to a stronger tumoricidal impact.

### 2.8 In Vivo ESCC Cancer Suppression Efficiency

**Figure 8:**
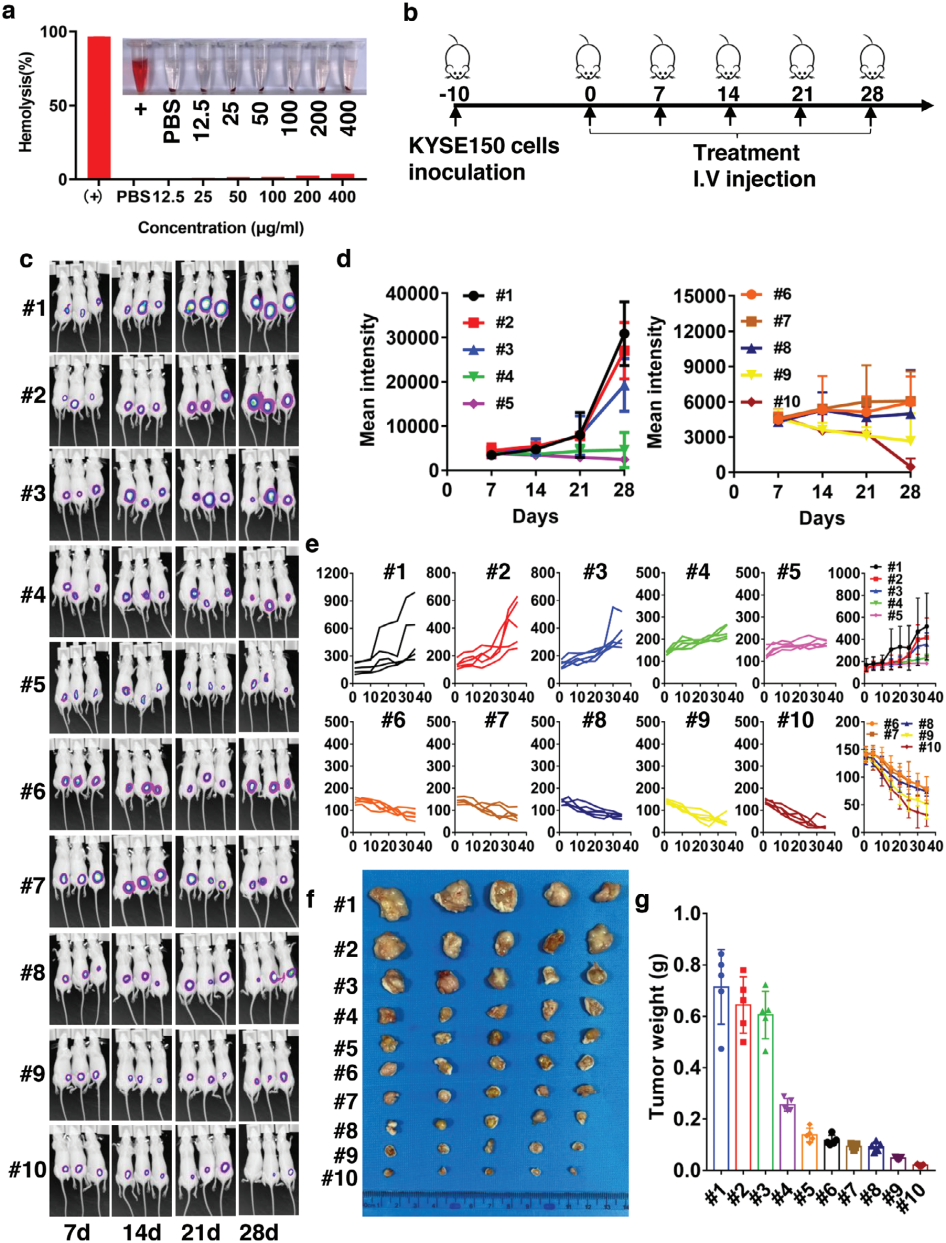
In vivo ESCC cancer suppression efficiency of EA1‐EYLNs‐PTX/siEFNA1 in KYSE‐150 tumor‐bearing mice. a) Hemolysis test of EA1‐EYLNs‐PTX/siEFNA1 at different concentrations. b) Schematic diagram of therapeutic treatment protocol in luciferase‐expressing KYSE‐150 tumor‐bearing mice. Treatment includes PBS (#1), EYLNs (5 nmol, #2), EYLNs‐EA1 (5 nmol, #3), EYLNs‐siEFNA1 (5 nmol, #4), EA1‐EYLNs‐siEFNA1 (5 nmol, #5), PTX (120 nmol, #6), EYLNs‐PTX (5 nmol EYLNs with 120 nmol PTX, #7), EA1‐EYLNs‐PTX (#8), EYLNs‐PTX/siEFNA1 (#9), and EA1‐EYLNs‐PTX/siEFNA1 (#10). c, d) Luciferase signals of ESCC tumors in luciferase‐expressing KYSE‐150 tumor‐bearing mice. Luciferase signals were quantified every week for 4 weeks. e) Relative tumor volume changes measured and calculated every 3 d of luciferase‐expressing KYSE‐150 tumor‐bearing mice under different treatments. f, g) Tumor images and tumor weights of isolated tumor tissues in different NDDS‐treated groups.

Hemolysis, the breaking apart of red blood cells, can result in conditions such as anemia, jaundice, and other complications. It is essential to assess all intravenous drugs for potential hemolytic effects to guarantee safe administration. To this end, we incubated various concentrations of EA1‐EYLN‐PTX/siEFNA1 with peripheral blood cells. The hemolysis test validated the commendable blood compatibility of EA1‐EYLNs‐PTX/siEFNA1 (**Figure** **8**a). Notably, even as the concentration escalated, no significant hemolysis was detected. The hemolysis ratio remained below 2% at the peak concentration (400 µg mL^−1^), suggesting the nanomedicine's suitability for intravenous use in treating esophageal cancer.

A subcutaneous ESCC mouse tumor model was initially developed using luciferase‐tagged KYSE‐150 cells. Post 10 days of subcutaneous injection, tumor‐afflicted mice received intravenous dose of various solutions, including PBS (#1), EYLNs (5 nmol, #2), EYLNs‐EA1 (5 nmol, #3), EYLNs‐siEFNA1 (5 nmol, #4), EA1‐EYLNs‐siEFNA1 (5 nmol, #5), PTX (120 nmol, #6), EYLNs‐PTX (5 nmol EYLNs with 120 nmol PTX, #7), EA1‐EYLNs‐PTX (#8), EYLNs‐PTX/siEFNA1 (#9), and EA1‐EYLNs‐PTX/siEFNA1 (#10), at weekly intervals for five sessions (Figure 8b).

On the 35th day, post the quintuple treatment, there was a noticeable reduction in the tumor size in mice treated with EYLNs‐siEFNA1. Their tumor volume grew by merely 100 mm^3^, in contrast to PBS (350 mm^3^), marking a significant difference (Figure 8c–e). This suggests siEFNA1's potential to curtail tumor expansion. Observations from Figure 8c–g highlight that compared to other treatments, the combination of PTX and siEFNA1 in EYLNs was the most effective, leading to a minimal tumor growth. Crucially, introducing the EA1 modification further optimized this inhibitory effect. While EYLN‐PTX/siEFNA1 was already efficacious in stemming tumor growth, the EA1‐modified version reduced both tumor volume (Figure 8e,f) and weight (Figure 8g) by an additional estimated 50%. This indicates a pronounced advantage in combined therapy over singular treatments. Additionally, tumor tissues were harvested and stained with transferase dUTP nick‐end labeling (TUNEL) and immunohistochemistry (IHC) of Ki67, the specific staining images and quantification of TUNEL (Figure S4a,b, Supporting Information) and Ki67 (Figure S4c,d, Supporting Information) demonstrated that EA1‐EYLNs‐PTX/siEFNA1 greatly inhibited proliferation and promoted cellular apoptosis. The pronounced apoptosis induction efficiency in mice treated with EA1‐EYLNs‐PTX/siEFNA1 showcases its potent anti‐cancer and growth‐inhibiting properties. This aligns well with the positive outcomes observed in the in vitro antitumor efficacy assessments. Furthermore, PTX‐induced hepatotoxicity was observed by elevated ALT and AST indexes, the EA1‐EYLNs‐PTX/siEFNA1 showed an obvious liver protection effect, which reflected by decreased ALT and AST (Figure S5a, Supporting Information). Additionally, there were no marked differences in biochemical indexes (CK, CREA, and UREA, Figure S5b, Supporting Information), which reflect nephrotoxicity and cardiotoxicity. Histological examination of organ sections also suggested no obvious toxicities to liver and kidney tissues after treatment with EA1‐EYLNs‐PTX/siEFNA1 via intravenous administration (Figure S5c, Supporting Information). The aforementioned data indicated that the high biocompatibility of EA1‐EYLNs‐PTX/siEFNA1.

**Figure 9:**
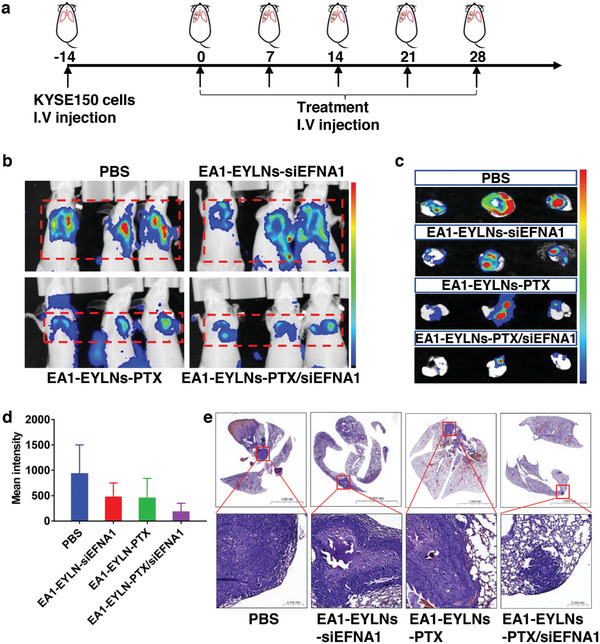
In vivo ESCC cancer suppression efficiency of EA‐EYLNs‐PTX/siEFNA1 in KYSE‐150 derived lung metastasis mice. a) Schematic diagram of therapeutic treatment protocol in BALB/c nude mice with KYSE‐150 derived lung metastasis. Treatments includes PBS, EA1‐EYLNs‐siEFNA1, EA1‐EYLNs‐PTX, and EA1‐EYLNs‐PTX/siEFNA1. b–d) Luciferase signals of lung metastasis in vivo (b) and isolated lung (c‐d) after 5 times of treatments. e) HE staining of lung metastasis sections. Scale bar: 5000 mm and 0.2 mm.

The efficacy of nanomedicine against ESCC with lung metastasis was further evaluated. Prior proteomic and in vitro cytological studies demonstrated that EFNA1 was markedly upregulated in ESCC tissue with lung metastasis, correlating positively with patient outcomes. Importantly, suppressing EFNA1 hindered ESCC cell movement, invasion, and the propensity for lung metastasis in mice. To delve deeper into EFNA1's potential as a therapeutic target, mice with lung‐involved ESCC received different nanomedicine regimens, administered weekly for five sessions (**Figure** **9**a). The data revealed that EA1‐guided PTX delivery curtailed lung metastasis, with targeted siEFNA1 exhibiting even greater efficacy than PTX alone. The combination of siEFNA1 and PTX showcased the most significant reduction in lung metastasis, affirming their synergistic therapeutic impact (Figure 9b‐e).

Given these outcomes, EFNA1 emerges as a promising molecular target for ESCC treatment. The potency of EA1‐mediated ESCC‐specific nanomedicine was convincingly demonstrated, introducing an innovative targeted therapeutic approach for ESCC. Nonetheless, the intricate molecular mechanisms through which EFNA1 influences ESCC progression partially uncharted, and certain constraints persist. Recognizing that tumor advancements is multifactorial, multiple molecules potentially liked to lung‐involved ESCC were pinpointed. Yet, this investigation primarily focused on gauging the efficacy of combining EFNA1 and PTX in treating ESCC, both with and without lung metastasis. Future research should prioritize uncovering additional potent molecular targets for ESCC treatment.

## 3 Conclusion

EFNA1 stands out as a pivotal factor linked to the lung metastasis of ESCC. The chosen aptamer, EA1, exhibited robust affinity and specificity for ESCC cells. Through EA1, precise co‐delivery of siRNA and chemotherapy was achieved, creating a synergistic treatment avenue for ESCC. Both in vitro and in vivo findings underscores the significant clinical potential of EA1‐EYLNs‐PTX/siEFNA1.

## 4 Experimental Section

### Materials

Paclitaxel was sourced from MCE, and siEFNA1 was synthesized by GenePharma. Polar lipid extract kits were purchased from CELL BIOLABS, while PKH26 was acquired from GOYOO. Yeasen Biotech supplied LysoTracker green, and the PKH67 red fluorescent cell linker kit and branched poly‐ethylenimine (PEI) were from Sigma. DiR dye was products of US EVERBRIGHT. The anti‐EFNA1 antibody was procured from Abcam, HRP‐conjugated goat anti‐rabbit IgG, and CoraLite 488‐conjugated goat anti‐rabbit IgG were from Proteintech. Matrigel was a product of Corning, RNase A from LEAGENE, and D‐luciferin potassium salt came from PerkinElmer. Proteinase K was obtained from AXYGEN, crystal violet staining solution from KeyGEN Biotech, and 4,6‐amidine‐2‐phenylindole dihydrochloride (DAPI) was sourced from Southern Biotech.

### Cell Lines

The human esophageal squamous cell carcinoma KYSE‐150 was maintained in the laboratory and authenticated by the China Center for Type Culture Collection (CCTCC). The luciferase‐expressing KYSE‐150 cell (Luc‐KYSE‐150) was developed in‐house. Other human esophageal cancer cell lines (Eca9706, KYSE30, KYSE410), the human colorectal cancer line SW620, the human tongue squamous cell carcinoma (CAL‐27), the human pharyngeal squamous cell carcinoma (FaDu), the human lung adenocarcinoma line A549, the human epidermoid carcinoma line A431, and the human acute lymphoblastic leukemia cell line Nalm‐6 were all procured from the Cell Bank of the Chinese Academy of Sciences (Shanghai, China). The HEEC line was sourced from Procell Life Science & Technology Co., Ltd. All cell lines were cultivated under humid conditions at 37 °C with a 5% CO_2_ atmosphere. The culture medium for KYSE‐150, Eca9706, KYSE30, KYSE410, CAL‐27, FaDu, A549, and A431 consisted of DMEM with 10% FBS and antibiotics (100 µL/ml streptomycin and 100 µL ml^−1^ penicillin). In contrast, SW620, HEEC, and Nalm‐6 were maintained in Leibovitz's L‐15, MEM with NEAA, and RPMI1640, respectively.

### Animals

Female BALB/c nude mice (SPF, aged 3–4 weeks, license number: NO.202219987) were sourced from GemPharmatech Co., Ltd. (Nanjing, China). All animal studies received approval from the Animal Care and Use Committee of The Affiliated Huai'an No.1 People's Hospital of Nanjing Medical University (DW‐P‐2023‐001‐07).

### Esophageal Squamous Cell Carcinoma Tissue Specimens

ESCC diagnoses were confirmed through pathology post‐endoscopic biopsy at the Department of Thoracic Surgery, The Affiliated Huai'an No.1 People's Hospital of Nanjing Medical University. Tumor tissues of 184 were obtained (ranging from highly to poorly differentiated ESCC) and adjacent normal esophageal samples from the ESCC Biobank of the same institution (Jan 2012 – Oct 2015), in line with the HIPAA protocol. The associated tissue study received approval from the ethical committee of the hospital (KY‐2023‐053‐01). All participants, or their relatives, provided either written informed consent or oral permission. The Kaplan‐Meier method was used to plot the overall survival curve, based on EFNA1's relative expression and its cut‐off value.

### Differential Protein Screening by Liquid Chromatography‐Tandem Mass Spectrometry (LC‐MS/MS)

Proteins from ESCC and neighboring tissues were extracted using a protein extraction kit (Thermo Fisher, CA, USA). For differential protein identification and quantification, LC‐MS/MS was performed using Q Exactive Plus (Thermo Fisher Scientific). The Proteome Discoverer platform (Version 2.1, Thermo) was employed for data processing, with MASCOT 2.6 as the search engine.

### Bioinformatics of EFNA1 Expression from a Public Database

EFNA1 expression across 5 normal tissues, 169 adenocarcinoma tissues, and 169 squamous cell carcinoma tissues was analyzed using The Cancer Genome Atlas (TCGA) database (https://cancergenome.nih.gov/).

### EFNA1 mRNA Expression in ESCC Tissue and Cells

EFNA1 expression in ESCC tissues and cells was determined by RT‐qPCR. In brief, total RNA from tissue or cell samples was extracted using the TRIzol reagent (Thermo Fisher, CA, USA), with samples stored at −80 °C until analyzed. The quality of the extracted RNA was assessed with the NanoDrop 2000 spectrophotometer (NanoDrop, Wilmington, DE). RNA samples were then reverse transcribed using Takara kits (RR047A Takara PrimeScript RT reagent Kit with gDNA Eraser, Japan) as per the manufacturer's guidelines. The Roche LightCycler 480 Realtime system with Universal SYBR Green Master Mix (Vazyme, China) quantified EFNA1 mRNA levels. Primers used were listed in **Table** **3**.

**Table 3:** Primer sequences used for EFNA1 mRNA determination.

Gene	Context sequence (5′‐3′)	Amplification product
EFNA1	Forward: 5′‐CAGCGCTTCACACCTTTCAC‐3′	79 bp
	Reverse: 5′‐GGTGGATGGGTTTGGAGATGT‐3′	
GAPDH	Forward: 5′‐CTGCACCACCAACTGCTTAG‐3′	76 bp
	Reverse: 5′‐AGGTCCACCACTGACACGTT‐3′	

### Immunohistochemistry (IHC) Staining for Determination of EFNA1 Expression

Slides were examined by pathologists before proceeding. The ESCC and paired adjacent tissues were fixed in 4% paraformaldehyde (PFA) overnight at room temperature and then processed with xylene for paraffin embedding. Sectioned slices (5 µm) underwent dehydration by being twice dewaxed in xylene for 15 min and then rehydrated in graded ethanol (100%, 95%, 85%, 70%) for 2 min each. The sections were rinsed with PBS and submerged in boiled sodium citrate buffer (pH6.2) for 30 min for retrieval. After this, the sections were blocked with 1% BSA for 30 min at room temperature. Following removal of the blocking solution, sections were incubated with primary antibody (anti‐EFNA1 rabbit antibody, 1:500 5D4Q5, Invitrogen, USA) overnight at 4˚C. After washing thrice with TBST, the sections were exposed to goat anti‐rabbit IgG HRP‐conjugated secondary antibody (1:1000, Abcam, USA) for 1 h at 37˚C. Sections were then stained with diaminobenzidine (Dako) and counterstained with hematoxylin. Images were captured using an upright fluorescence microscope (Eclipse Ni‐U, Nikon‐Japan). The H‐score was utilized for quantification.

### Transient Transfection using siRNA Interference

For ESCC cell lines' transient transfection, two EFNA1 siRNA and negative control sequences of EFNA1 (summarized in **Table** **4**) using Lipofectamine transfection reagent were designed and purchased from GenePharma. KYSE‐150 cells (in exponential growth) were seeded into 6‐well culturing plates at 6 × 10^5^ cells well^−1^. After a 24 h incubation, cells underwent transfection with siRNA using Lipofectamine 3000 reagent (Thermo Fisher, CA, USA) for 6 h. After 48 h incubation, total RNA and protein of the cells were extracted for evaluating EFNA1 mRNA expression as previously described and for Western blot analysis using anti‐EFNA1 antibody, respectively. Concurrently, CCK‐8 and colony‐formation assays were conducted to assess the impact of EFNA1 on the proliferation of transfected KYSE‐150 cells.

**Table 4:** siRNA senses for EFNA1 RNA interference.

Name	Context sequence (5′‐3′)
si‐negative control	sense: 5′‐UUCUCCGAACGUGUCACGUTT‐3′
	antisense: 5′‐ACGUGACACGUUCGGAGAATT‐3′
si‐EFNA1‐1	sense: 5′‐UGCCUUUAAGCCAAAGAAATT‐3′
	antisense: 5′‐UUUCUUUGGCUUAAAGGCAGG‐3′
si‐EFNA1‐2	sense: 5′‐UCUUCUGGAACAGUUCAAATT‐3′
	antisense: 5′‐UUUGAACUGUUCCAGAAGACG‐3′

### Lentivirus Plasmid Construction and Establishment of Stable Cell Lines

For the stable silencing of the EFNA1 gene, a lentivirus plasmid was constructed using the BR‑V‑108 vector. Using the EFNA1 gene as a template, the RNAi target sequences for EFNA1 (outlined in **Table** **5**) and their corresponding single‑stranded DNA oligos were designed and synthesized by Generay Biotech. Annealing at 90˚C for 15 min produced the double‐stranded DNA oligo. After linearizing the BR‑V‑108 vector with *AgeI* and *EcoRI* restriction sites, target sequences were integrated into the BR‑V‑108 vector (100 ng µL^−1^) using T4 DNA Ligase. The ligation product was then introduced into 100 µL TOP10E. coli competent cells (Tiangen, Beijing, China) and allowed to grow in antibiotic‐free LB liquid medium at 37˚C for 1 h. Afterward, LB solid medium (with Amp) inoculated with 150 µL of the bacterial solution was cultivated overnight at 37˚C. Individual colonies were isolated with a sterile tip and verified through PCR amplification. Positive recombinant plasmids were proliferated in 150 mL LB liquid medium and then isolated using the EndoFree Maxi Plasmid Kit (DP117, Tiangen, Beijing, China). Verification of the amplified recombinant plasmids was achieved through double enzyme digestion and gel electrophoresis. High‐quality recombinant plasmids were then introduced into HEK‐293T cells for virus production. After 72 h, the cell supernatant was harvested, and the lentivirus quality, MOI, and transfection duration were assessed. A transfection time of 72 h was selected for subsequent procedures.

**Table 5:** shRNA senses for EFNA1 RNA interference.

Name	Context sequence (5′‐3′)
sh‐negative control	sense: 5′‐UUCUCCGAACGUGUCACGUTT‐3′
	antisense: 5′‐ACGUGACACGUUCGGAGAATT‐3′
sh‐EFNA1‐1	sense: 5′‐UGCCUUUAAGCCAAAGAAATT‐3′
	antisense: 5′‐UUUCUUUGGCUUAAAGGCAGG‐3′
sh‐EFNA1‐2	sense: 5′‐UCUUCUGGAACAGUUCAAATT‐3′
	antisense: 5′‐UUUGAACUCUUCCAGAAGACG‐3′

To generate stable cell lines, KYSE‐150 cells (during their exponential growth phase) were plated into 6‐well culture plates at 2 × 10^5^ cells well^−1^. Following a 24 h incubation, 20 µL of lentivirus (1 × 10^8^ TU mL^−1^) was added to achieve cell transfection. Three days post‐transfection, both transfection and knockdown efficiencies were measured using qRT‐PCR and Western blot analyses. RTCA assay and a colony‐formation test were conducted to validate the effects of EFNA1 silencing on the growth of shEFNA1‐transfected KYSE‐150 cells. Additionally, the wound‐healing assay, along with cell migration and invasion tests using Transwell, were executed to ascertain the impact of EFNA1 silencing on cell migratory capabilities.

### Western Blotting

siEFNA1‐transfected, stably shEFNA1‐transfected, and various NDDS, including PBS (#1), EYLNs (5 nmol, #2), EYLNs‐EA1 (5 nmol, #3), EYLNs‐siEFNA1 (5 nmol, #4), EA1‐EYLNs‐siEFNA1 (5 nmol, #5), PTX (120 nmol, #6), EYLNs‐PTX (5 nmol EYLNs with 120 nmol PTX, #7), EA1‐EYLNs‐PTX (#8), EYLNs‐PTX/siEFNA1 (#9), and EA1‐EYLNs‐PTX/siEFNA1 (#10) treated KYSE‐150 cells were lysed with RIPA lysis buffer (containing 1 mM PMSF and 1 mM cocktail) on ice for 30 min. The lysate was then centrifuged at 4 °C for 20 min at 12 000 g to obtain the supernatant containing total cellular proteins. The BCA protein assay (Beyotime, Shanghai, China) determined protein concentrations. After denaturing the proteins with 5 × loading buffer at 100˚C, they were separated using sodium dodecyl sulfate‐polyacrylamide gel electrophoresis (SDS‐PAGE) and subsequently transferred to PVDF membranes (Millipore, USA). Membranes were blocked with 5% BSA at room temperature for 1 h. Next, they were incubated overnight at 4˚C with the primary antibody (anti‐EFNA1 rabbit antibody, 1:1000 5D4Q5, Invitrogen, USA). After washing with TBST, membranes were incubated with HRP‐conjugated secondary antibody for 2 h at room temperature. The relative expression of EFNA1 protein was visualized using ECL reagent (ZETA LIFE, USA) and quantified with Image J.

### CCK‐8 Assays

siEFNA1‐transfected KYSE‐150 cells were plated in 96‐well plates at a density of 2 × 10^3^ cells well^−1^ in 100 µL. After incubation for 24 h, 48 h, and 72 h, the cells were treated with CCK8 solution (10 µL well^−1^) and incubated for 2 h at 37˚C. the OD_490 nm_ was measured using a microplate reader (Epoch, BioTek, USA) to assess cell viability.

### Colony‐Formation Assay

KYSE‐150 cells, both siEFNA1 and shEFNA1‐transfected, were plated in 6‐well plates at a density of 2 × 10^3^ cells well^−1^ in 2000 µL. For siEFNA1 cells, transfection was done using Lipofectamine 3000. Similarly, shEFNA1‐transfected KYSE‐150 cells were collected, digested, resuspended, and seeded into 6‐well plates. After 14 days of culture, cell were fixed with 4% PFA and stained with 0.1% crystal violet. Colonies consisting of more than 50 cells were visualized, captured, and quantified using Image J.

### Real‐Time Cell Analysis (RTCA)

RTCA was employed to evaluate the cell viability of both shEFNA1‐transfected and various NDDS‐treated KYSE‐150 cells. In brief, KYSE‐150 cells transfected with shEFNA1 and the negative control shRNA were plated in E‐plates (2 × 10^3^ cells well^−1^, 100 µL) and incubated at 37 °C for 72 h. Cell growth curves were continuously monitored using the xCELLigence System's Real‐Time Cell Analyzers (Agilent, USA). For assessing the cytotoxicity of different NDDS treatments, KYSE‐150 cells (1 × 10^4^ cells well^−1^, 150 µL) were incubated overnight at 37 °C in E‐plates. Post incubation, cells were exposed to various treatments including PBS, EYLNs, EA1‐EYLNs, EYLNs‐siEFNA1, EA1‐EYLNs‐siEFNA1, PTX, EYLNs‐PTX, EA1‐EYLNs‐PTX, EYLNs‐PTX/siEFNA1, and EA1‐EYLNs‐PTX/siEFNA1 for 72 h. Cell growth was subsequently recorded via RTCA.

### Cell Migration and Invasion

The migratory and invasive capabilities of shEFNA1‐transfected and various NDDS‐treated KYSE‐150 cells were evaluated using the wound‐healing and Transwell assays. For the wound‐healing assay, KYSE‐150 cells, either transfected with shEFNA1 or the negative control (6x10^5^ cells well^−1^, 2000 µL), were cultured in 6‐well plates at 37 °C. Post a 24 h incubation, once the monolayer cells reached confluency, a scratch was introduced using a 20 µL pipette tip. and the cells were then gently washed thrice with PBS and further cultivated in DMEM medium supplemented with 0.5% FBS for an additional 36 h. Snapshots of the wound were taken at 0 h, 12 h, 24 h, and 36 h intervals using a phase‐contrast microscope (Leica, USA). The wound dimensions were analyzed with ImageJ employing the Wound_healing_size_tool. The degree of wound closure was quantified as healing percentage (% of scar = healing area / initial area × 100%).

For the Transwell assay, KYSE‐150 cells transfected with either shEFNA1 or the negative control (2x10^4^ cells well^−1^, 200 µL) were suspended in basic DMEM and seeded into the upper chambers of Transwell inserts (with 8 mm pore size, NEST, WuXi, China) placed in a 24‐well plate. DMEM medium enriched with 10% FBS was introduced into the lower chambers. After 48‐h, cells on the membrane's upper surface were wiped away using a cotton swab. In contrast, those adhering to the membrane's lower surface were fixed using 4% PFA and stained with 0.1% crystal violet. Images of invaded cells were taken across five random fields, and their numbers were determined using Image J.

To evaluate the inhibition of NDDS on ESCC cell migration, 3x10^4^ KYSE‐150 cells were cultured in Transwell chambers. They were then treated with various NDDS for 48 h. Subsequently, stained cells that penetrated were quantified. Invasion assays utilized Matrigel‐coated Transwell inserts, adhering to the above‐mentioned procedure.

### DNA Library and Primers

The study's ssDNA library (sequence: 5′‐AGCCTAAGCCTGTCCAGGAATCG‐N32‐ATGGCTTAGTGGCACGATTAGGTC‐3′, 79 nucleotides) comprises two primer regions (at 5′ and 3′ ends) for PCR and a 32 nt random region, which was developed with equal incorporation of A, T, C, and G. For library preparation, the forward primer was tagged with carboxylfluorescein (FAM) at its 5′ end, denoted as 5′‑FAM‑AGCCTAAGCCTGTCCAGGAATCG‑3′. This was for determining binding via flow cytometry (B6‐plus, BD Bioscience). The reverse primer, labeled with biotin at the 5′ end, was used for separating the PCR products with streptavidin‐coated sepharose beads, represented as 5′‑Biotin‑GACCTAATCGTGCCACTAAGCCAT‑3′. GenePharma synthesized and purified the entire ssDNA library and the primers.

### The Cell‐SELEX Process

As depicted in Figure S2a (Supporting Information), the cell‐SELEX process was executed. For positive selection, the initial ssDNA library, in binding buffer (4.5 g L^−1^ glucose, 5 mM MgCL_2_, 1 mg mL^−1^ BSA, 0.1 mg mL^−1^ yeast tRNA), was denatured at 95 °C for 5 min, and promptly cooled on ice for 10 min. It was then incubated with KYSE‐150 cells at 4 °C for 60 min. Post three washes with the washing buffer (4.5 g L^−1^ glucose, 5 mM MgCL_2_), 500 µL ddH_2_O was introduced to the cells. These cells were then heated at 95 °C for 10 min to extract the bound ssDNA. The harvested ssDNA underwent PCR amplification, employing the specified forward and reverse primers. The PCR entailed: 3 min of predenaturation at 95˚C, 15 s of denaturation at 95˚C, annealing, 90 s of extension at 72 °C, repeated for 35 cycles, and a final 10 min extension at 72 °C. The PCR products were then exposed to streptavidin‐modified sepharose beads at room temperature for 30 min. Denaturation was initiated with 200 mM NaOH for 10 min, followed by centrifugation. Last, the acquired ssDNA library was desalted, measured, and dried for subsequent selection rounds.

Following three rounds of positive selection, HEEC cells were introduced for negative selection. The ssDNA library, at a concentration of 2.5 µM in binding buffer, was incubated with these HEEC cells at 4 °C for 30 min. Subsequently, the supernatant containing unbound DNA was gathered for the subsequent round of selection. Throughout this process, screening intensities were progressively escalated. This involved decreasing the positive selection incubation duration from 30 to 60 min, extending the negative selection incubation from 30 to 60 min and augmenting the washing cycles from 3 to 5 rounds. Following 20 selection cycles, the ssDNAs samples were amplified and integrated into the pUC19 vector via Escherichia coli DH5a cells. Chosen clones, namely EA1, EA2, EA3, EA4, and EA5underwent sequencing their secondary structures were scrutinized with DNAMAN v.3.2 software.

### Flow Cytometry Assays

Flow cytometry assays were utilized to observe library enhancement and to contrast aptamer binding affinities. In brief, ssDNA and the chosen aptamers (250 nM, from EA1 to EA5), labeled with Cy5.5, were incubated alongside KYSE‐150 cells in binding buffer at 4 °C for 60 min. Post three washes, cells were reconstituted in 400 µL binding buffer, and fluorescence readings (EX 488 nm and EM 525 nm bandpass channel) were acquired via flow cytometry analyzing 10 000 events. A random DNA sequence served as the control.

To determine the binding potencies of EA1 and EA2, various concentrations (ranging from 0 nM, 50 nM, 100 nM, 150 nM, 200 nM, 250 nM, 300 nM) of Cy5.5 tagged EA1 or EA2 were combined with KYSE‐150 cells and incubated at 4 °C for 60 min. Post a triple wash in washing buffer, cells were resuspended in 400 µL binding buffer to proceed with flow cytometry. The aptamers' dissociation constants (K_d_) were deduced using the one‐site saturation equation, Y = B_max_ X/(K_d_ + X), interpreted via the Origin software.

To assess the binding specificity of the identified aptamers, both ESCC cell lines (KYSE‐30, KYSE‐410, ECa9706) and other tumor cell lines (SW620, CAL‐27, FaDu, A‐431, A549, and Nalm‐6) were used, along with leukocytes from a healthy volunteer. These cells were exposed to 250 nM of Cy5.5 labeled EA1 or EA2 at 4 °C for 60 min. Post incubation, cells underwent two washes with washing buffer, and after resuspension in 400 µL binding buffer, the aptamer signal present on the cells was determined using flow cytometry.

To assess the apoptosis levels of NDDS‐treated cells, KYSE‐150 cells were seeded into 6‐well plates with 2 × 10^5^ cells per well and incubated at 37 °C overnight. Cells were washed with PBS and incubated for another 24 h with free drugs or various NDDS. After incubation, cells were collected and stained with the Annexin V‐FITC apoptosis detection kit following the manufacturer's protocols. The stained cells were analyzed by FCM.

### Confocal Imaging

To validate the aptamer's binding capacity, both KYSE‐150 (4 × 10^4^ cells) and HEEC cells (4 × 10^4^ cells) were allotted to confocal dishes and incubated for 24 h. Following a triple wash with PBS, cells were exposed to 250 nM FAM‐tagged aptamers EA1 and EA2 in a binding buffer at 4 °C for 1 h. After a wash in washing buffer, the cells were visualized using an LSCM (Nikon, Japan).

To corroborate the suitability of EA1 with clinical tissue samples, various tumor tissue sections, such as ESCC, gastric cancer, liver cancer, colon cancer, and lung cancer, alongside corresponding adjacent tissues underwent dewaxing, rehydration, and antigen retrieval. Subsequently, the sections were blocked using 500 nM random sequences for 30 min. After three PBS washes, sections were exposed to Cy5.5 labeled aptamer EA1 and stained with DAPI for another 30 min. The resultant fluorescence images were captured using a laser scanning confocal microscope.

### Development of Nano‐Delivery Systems

Initially, polar lipids were extracted from egg yolks using a kit designed for polar/neutral lipid separation, then natural EYLN was prepared following the guidelines from the previous research.^[^ ^14^, ^15^, ^16^ ^]^ To formulate PTX‐loaded EYLNs (EYLNs‐PTX), Extracted lipids (3 mg) and PTX (120 nmol) in chloroform were dissolved, achieving a clear solution through ultrasonic treatment. The solution was then dried using nitrogen gas to produce a thin film complex. This complex was hydrated with 400 µL PBS and subjected to sonication for 20 min. Centrifugation at 100 000 g for 30 min was used to remove any unencapsulated PTX.

For the creation of PTX/siEFNA1‐loaded EYLNs (EYLNs‐PTX/siEFNA1), EYLNs were combined with PEI (33 µg) and shaken at room temperature for 1 h. After adding siEFNA1 (5 nmol) and shaking for another 30 min, the mixture was sonicated in intervals: every 2 min thrice. To prepare EA1‐modified PTX/siEFNA1‐loaded EYLNs (EA1‐EYLNs‐PTX/siEFNA1), EYLNs were mixed with PEI (144 µg), shaken at room temperature for 1 h, and then combined with siEFNA1 (1.8 nmol) for an additional 30 min shaking period. This mixture underwent the same sonication pattern. Aptamer EA1 (4.5 nmol) was then combined with EYLNs‐PTX/siEFNA1, shaken for 30 min at room temperature, and sonicated as previously mentioned. The formulations of EA1‐EYLNs, EYLNs‐siEFNA1, EA1‐EYLNs‐siEFNA1, and EA1‐EYLNs‐PTX followed the same aforementioned protocol. All crafted NDDS were preserved at 4 °C for subsequent examinations.

### siRNA and Aptamer Binding Efficiency

To determine the binding efficiency of siEFNA1 and EA1 to EYLNs, both EA1‐EYLNs‐PTX/siEFNA1 and EYLNs‐PTX/siEFNA1 were run on a 2.0% agarose gel. EA1 and siEFNA1 served as controls. The visualized DNA ladders were captured using a Bioimage system (UVP, GelDoc‐It2 Imager).

### Characterization of Nano‐delivery Systems

The characterization of the nano‐delivery systems involved evaluating aspects such as entrapment efficiency, drug loading, morphology, particle size, ζ potential, and storage stability of EA1‐EYLNs‐PTX/siEFNA1. Additionally, an enzymatic stability assay was conducted. To determine entrapment efficiency (EE%) and drug loading (DL%), the non‐encapsulated PTX was separated through a cellulose nitrate membrane. The PTX content was quantified using HPLC (Agilent 1260 liquid chromatography system, Agilent, USA) with a Waters symmetry C18 column (4.6 × 150 mm, 3.5 µm, Waters, Milford, MA, USA) set at 25 °C. The DAD wavelength was established at 227 nm, while the mobile phase consisted of methanol and H_2_O (75:25, v/v) flowing at 1.0 mL mi^−1^n. The HPLC method was validated for its specificity, linearity, accuracy, and precision. All assays were carried out in triplicate. EE% and DL% were computed as follows: EE% = (1‐W_non‐encapsulated_/W_total_) × 100%, DL% = (W_total_‐W_non‐encapsulated_)/(W_total_‐W_non‐encapsulated_ + W_exc_) × 100%.

For morphological studies, the negative‐staining technique was employed. A droplet of the diluted solutions of both EA1‐EYLNs‐PTX/siEFNA1 and EYLNs‐PTX/siEFNA1 was deposited on a copper grid, which was then stained with a 2% acidic phosphotungstic solution for 30 s. After drying these prepared thin films for 30 min, they were inspected under a TEM, FEI Tecnai G2 Spirit BioTwin).

Particle size and ζ potential were gauged using the PSS Nicomp 380 Z3000 instrument, which assessed the PDI, and the ζ potential of EA1‐EYLNs‐PTX/siEFNA1 and EYLNs‐PTX/siEFNA1 based on dynamic light scattering and electrophoretic mobility. Samples were diluted, placed in cuvettes, and measurements were taken at a 90° angle and 25 °C. These assessments were done in triplicate.

For storage stability evaluation, samples of EA1‐EYLNs‐PTX/siEFNA1 and EYLNs‐PTX/siEFNA1 were preserved in PBS containing 10% FBS, either at 4 °C or 37 °C. At specific time intervals (1, 3, 7, 14 days), measurements of particle size and ζ potential were taken.

For enzymatic stability assay, EA1‐EYLNs‐PTX/siEFNA1 was subjected to incubation with RNase (0.1 µg µL^−1^) at 37 °C over varying durations (0, 0.5, 1, 2, 3, and 6 h). The siEFNA1 intensity was determined using 2% agarose gel electrophoresis, with quantification achieved via Image J.

### In Vitro Drug Release

The release behavior of EA1‐EYLNs‐PTX/siEFNA1 was analyzed using the dialysis method. Specifically, 1 mL of EA1‐EYLNs‐PTX/siEFNA1 was placed into dialysis bags (Mol_wt._ = 8000 Da, 25 mm × 5 m, Spectrum Medical Industries Inc., USA). These bags were then submerged in varied dissolution media (pH 7.4, pH 5.5, 50 mL each) and agitated at 100 rpm at 37 °C. At set intervals (0.5, 1, 3, 6, 12, 24, 36, 48, 60, and 72 h), 1 mL samples were extracted and immediately replaced with 1 mL of pre‐warmed dissolution media. The released PTX content was measured using the previously described HPLC method, leading to the creation of in vitro cumulative drug release profiles.

### Cellular Uptake and Endosomal Escape

KYSE‐150 cells (4 × 10^4^ cells) were placed in confocal dishes for a 24 h incubation. After three PBS washes, these cells were treated with PKH26‐labeled EYLNs‐PTX/siEFNA1 or EA1‐EYLNs‐PTX/siEFNA1 at 37 °C for 6 h. Following another PBS wash, the cells were fixed using 4% PFA. Nuclei were stained with DAPI (20 mg mL^−1^) for 10 min in a 37 °C dark environment. Ultimately, cell images were captured, and the PKH26 signal intensity was documented using an LSCM (Nikon, JAPAN).

For pinpointing the cellular localization of EA1‐EYLNs‐PTX/siEFNA1, post‐treatment of cells with PKH67‐labeled variants for 2 h, 4 h, 6 h, and 12 h respectively, cells underwent three PBS washes and were then stained with LysoTracker Red (2 µM) for 1 h and DAPI for 10 min. The cellular images were subsequently taken using a laser scanning confocal system.

For tumor penetration capacity, KYSE‐150 cell spheroids were formulated by mixing KYSE‐150 cells (1 × 10^3^ cells) with 150 µL of Matrigel (5 mg mL^−1^, diluted in DMEM) and then seeding them into a confocal dish. After a 14 d‐incubation, the spheroids were exposed to PKH26‐labeled EYLNs‐PTX/siEFNA1 or EA1‐EYLNs‐PTX/siEFNA1 for 24 h. Following three PBS washes, the spheroids were fixed using 4% PFA for 30 min, and stained with DAPI. The PKH26 signal intensity within the spheroids was then captured at depths of 8, 16, 24, 32, and 40 µm using a laser scanning confocal system.

### Hemolysis Test

Mouse blood was centrifuged at 5000 rpm for 5 min to isolate red blood cells (RBCs). The resuspended RBCs (50 µL) were then incubated with different concentrations of EA1‐EYLNs‐PTX/siEFNA1 (12.5, 25, 50, 100, 200, 400 µg mL^−1^) at 37 °C for 2 h. 1 mL of PBS served as a negative control, while 1% Triton‐X 100 was used as a positive control. After 2 h incubation, samples were centrifuged at 12,000 rpm for 5 min, and the absorbance of the supernatants at 450 nm was assessed using a microplate reader. The hemolysis ratio was subsequently determined.

### Xenograft Tumor Model

To evaluate the impact of EFNA1 on the growth of ESCC cells in vivo, shEFNA1‐transfected and control luciferase‐expressing KYSE‐150 cells were injected subcutaneously (5 × 10^6^/100 µL) and intravenously (1 × 10^6^/100 µL) into BALB/c nude mice. 14 d later, mice received an intraperitoneal injection of D‐luciferin (150 mg k^−1^g). Subcutaneous tumors derived from KYSE‐150 and lung metastases were visualized using a live imaging system (Bruker, FX Pro), with tumor fluorescence intensity recorded. After the final observation, the mice were euthanized, tumors excised, weighed, and photographed.

To study the anti‐cancer properties and biodistribution of the aptamer EA1 and various NDDS, another xenograft tumor model was established. For the subcutaneous tumor model, KYSE‐150 cells expressing luciferase (5 × 10^6^/100 µL) were injected subcutaneously into BALB/c nude mice. Once tumor volume reached ≈ 50 mm^3^, the mice were grouped randomly for further testing. For the lung metastasis model, luciferase‐expressing KYSE‐150 cells (1 × 10^6^/100 µL) were injected intravenously into BALB/c nude mice. The establishment of all mouse models was validated using the live imaging system.

### In Vivo Imaging

To assess the targeting capacity of aptamer EA1, Cy5.5‐labeled EA1 (5 nmol/100 µL) was intravenously administered to KYSE‐150 tumor‐bearing mice (both subcutaneous tumor and lung metastasis models). 30 min post‐injection, the EA1 distribution was visualized using a live imaging system. Subsequently, key organs (heart, liver, spleen, lung, kidney, and tumor) were extracted and further scanned using the same imaging system.

For the evaluation of in vivo biodistribution of aptamer‐modified EYLNs, preparations of Cy5.5‐labeled EA1‐EYLNs and Cy5.5‐labeled EA5‐EYLNs were made as described earlier and injected intravenously into KYSE‐150 tumor‐bearing mice. Live imaging was executed at specific intervals (60, 90, 120, and 150 min). After the final observation, the mice were euthanized, key organs were retrieved, and the distribution of aptamer‐modified EYLNs was both visualized and quantified.

### In Vivo Anti‐Cancer Effects

To ascertain the tumor‐suppressing impact of different NDDS, KYSE‐150 tumor‐bearing mice were allocated randomly into 10 groups: PBS, EYLNs (5 nmol), EYLNs‐EA1 (5 nmol), EYLNs‐siEFNA1 (5 nmol), EA1‐EYLNs‐siEFNA1 (5 nmol), PTX (120 nmol), EYLNs‐PTX (5 nmol EYLNs with 120 nmol PTX), EA1‐EYLNs‐PTX, EYLNs‐PTX/siEFNA1, and EA1‐EYLNs‐PTX/siEFNA1. Mice received intravenous treatments every 7 d for 5 times. Tumor dimensions and mouse weight were documented tri‐weekly, and luciferase signals from the tumors were captured weekly using the Bruker FX Pro imaging system. A week after the final dose, mice were euthanized, tumors excised, and photographed. HE staining and immunohistochemical (IHC) staining for Ki67 were conducted to evaluate cancer cell proliferation, while TUNEL staining was utilized to identify apoptotic cells.

To delve deeper into the inhibitory effects of EA1‐modified EYLNs on lung metastasis, BALB/c nude mice with KYSE‐150 induced lung metastasis received intravenous administrations of PBS, EA1‐EYLNs‐siEFNA1, EA1‐EYLNs‐PTX, and EA1‐EYLNs‐PTX/siEFNA1 every 7 days, totaling five doses. The metastatic tumors' progression was tracked using luciferase signal detection and HE staining.

### Statistical Analysis

Data processing and visualization were conducted using GraphPad Prism software (version 7.0). All data were shown as mean ± SD. Both one‐way and two‐way analyses of variance facilitated comparisons across multiple groups. An unpaired t‐test determined differences between two distinct groups. All findings were validated across a minimum of three independent trials. A *p‐value of less than 0.05 denoted statistical significance.

## Conflict of Interest

The authors declare no conflict of interest.

## Supporting information

Supporting Information

## Data Availability

The data that support the findings of this study are available on request from the corresponding author. The data are not publicly available due to privacy or ethical restrictions.

## References

[1] H. Sung , J. Ferlay , R. L. Siegel , M. Laversanne , I. Soerjomataram , A. Jemal , F. Bray , CA Cancer J. Clin. 2021, 71, 209. 33538338 10.3322/caac.21660

[2] D. Huo , X. Jiang , Y. Hu , Adv. Mater. 2020, 32, 1904337. 10.1002/adma.201904337 31663198

[3] J. Chen , X. Li , Y. Sun , Y. Hu , Y. Peng , Y. Li , G. Yin , H. Liu , J. Xu , S. Zhong , Chemistry 2017, 23, 17279. 28913948 10.1002/chem.201702945

[4] R. P. Singh , G. Sharma , A. P. Sonali , B. L. Pandey , B. Koch , M. S. Muthu , Int. J. Biol. Macromol. 2016, 83, 335. 26657586 10.1016/j.ijbiomac.2015.11.081

[5] Y. Hao , Y. Chen , X. He , R. Han , C. Yang , T. Liu , Y. Yang , Q. Liu , Z. Qian , Biomaterials 2023, 293, 121975. 36580720 10.1016/j.biomaterials.2022.121975

[6] E. Bolli , M. Scherger , S. M. Arnouk , A. R. Pombo Antunes , D. Straßburger , M. Urschbach , J. Stickdorn , K. De Vlaminck , K. Movahedi , H. J. Räder , S. Hernot , P. Besenius , J. A. Van Ginderachter , L. Nuhn , Adv. Sci. 2021, 8, 2004574. 10.1002/advs.202004574 PMC8132149 34026453

[7] M. C. Johnston , J. A. Nicoll , K. M. Redmond , P. Smyth , M. K. Greene , W. J. McDaid , D. K. W. Chan , N. Crawford , K. J. Stott , J. P. Fox , N. L. Straubinger , S. Roche , M. Clynes , R. M. Straubinger , D. B. Longley , C. J. Scott , J. Control Release 2020, 324, 610. 32504778 10.1016/j.jconrel.2020.05.046 PMC7429293

[8] R. Xu , K. Zhang , J. Liang , F. Gao , J. Li , F. Guan , Carbohydr. Polym. 2021, 261, 117846. 33766342 10.1016/j.carbpol.2021.117846

[9] Z. Zhang , W. Cheng , Y. Pan , L. Jia , J. Mater. Chem. B 2020, 8, 655. 31904073 10.1039/c9tb02284h

[10] P. Ding , Z. Wang , Z. Wu , Y. Zhou , N. Sun , R. Pei , ACS Appl. Mater. Interfaces 2020, 12, 20263. 32259427 10.1021/acsami.0c03355

[11] Z. Tang , C. Luo , Y. Jun , M. Yao , M. Zhang , K. He , L. Jin , J. Ma , S. Chen , S. Sun , M. Tao , L. Ding , X. Sun , X. Chen , L. Zhang , Y. Gao , Q. L. Wang , ACS Appl. Mater. Interfaces 2020, 12, 7984. 31971362 10.1021/acsami.9b22293

[12] A. D. Ellington , J. W. Szostak , Nature 1990, 346, 818. 1697402 10.1038/346818a0

[13] S. Ohuchi , Cell‐SELEX Technol. Biores. Open Access. 2012, 1, 265. 10.1089/biores.2012.0253 PMC3559206 23515081

[14] C. Tuerk , L. Gold , Science 1990, 249, 505. 2200121 10.1126/science.2200121

[15] Q. Wan , Z. Zeng , J. Qi , Z. Chen , X. Liu , Y. Zu , Mol. Ther. 2022, 30, 2242. 35143958 10.1016/j.ymthe.2022.02.004 PMC9171151

[16] T. Wang , Y. Luo , H. Lv , J. Wang , Y. Zhang , R. Pei , ACS Appl. Mater. Interfaces 2019, 11, 45455. 31718159 10.1021/acsami.9b16637

[17] M. Kim , J. S. Lee , W. Kim , J. H. Lee , B. H. Jun , K. S. Kim , D. E. Kim , J. Control Release 2022, 348, 893. 35760233 10.1016/j.jconrel.2022.06.039

[18] X. D. Cui , M. J. Lee , G. R. Yu , I. H. Kim , H. C. Yu , E. Y. Song , D. G. Kim , Int. J. Cancer 2010, 126, 940. 19642143 10.1002/ijc.24798

[19] Z. Gao , X. Han , Y. Zhu , H. Zhang , R. Tian , Z. Wang , Y. Cui , Z. Wang , R. Niu , F. Zhang , Cell Death Dis. 2021, 12, 414. 33879771 10.1038/s41419-021-03692-x PMC8058342

[20] P. C. Lee , S. T. Chen , T. C. Kuo , T. C. Lin , M. C. Lin , J. Huang , J. S. Hung , C. L. Hsu , H. F. Juan , P. H. Lee , M. C. Huang , Oncogene 2020, 39, 2724. 32005975 10.1038/s41388-020-1178-7 PMC7098884

[21] G. Castoria , F. Auricchio , A. Migliaccio , FASEB J. 2017, 31, 1289. 28031322 10.1096/fj.201601047R

[22] U. M. Mahajan , Q. Li , A. Alnatsha , J. Maas , M. Orth , S. H. Maier , J. Peterhansl , I. Regel , M. Sendler , P. R. Wagh , N. Mishra , Y. Xue , P. Allawadhi , G. Beyer , J. P. Kühn , T. Marshall , B. Appel , F. Lämmerhirt , C. Belka , S. Müller , F. U. Weiss , K. Lauber , M. M. Lerch , J. Mayerle , Gastroenterology 2021, 161, 996. 34097885 10.1053/j.gastro.2021.05.055

[23] M. W. Kim , H. Y. Jeong , S. J. Kang , I. H. Jeong , M. J. Choi , Y. M. You , C. S. Im , I. H. Song , T. S. Lee , J. S. Lee , A. Lee , Y. S. Park , Theranostics. 2019, 9, 837. 30809312 10.7150/thno.30228 PMC6376474

[24] Y. Shen , M. Li , T. Liu , J. Liu , Y. Xie , J. Zhang , S. Xu , H. Liu , Int. J. Nanomed. 2019, 14, 4029. 10.2147/IJN.S201688 PMC6549788 31213813

[25] L. Dai , G. Shen , Y. Wang , P. Yang , H. Wang , Z. Liu , J. Mater. Chem. B 2021, 9, 1151. 33434248 10.1039/d0tb02576c

[26] X. Li , L. Zhang , X. Guo , F. Xie , C. Shen , Y. Jun , C. Luo , L. Liu , X. Yu , Z. Zhang , Q. Wang , Y. Gao , K. Xu , J. Nanobiotechnol. 2021, 19, 388. 10.1186/s12951-021-01135-5 PMC8614048 34823537

[27] Y. Zhang , X. Chen , Y. Qiao , S. Yang , Z. Wang , M. Ji , K. Yin , J. Zhao , K. Liu , B. Yuan , Anal. Chem. 2022, 94, 17212. 36459499 10.1021/acs.analchem.2c03863

[28] X. Chen , Y. Zhang , Y. Shi , T. Niu , B. Li , L. Guo , Y. Qiao , J. Zhao , B. Yuan , K. Liu , Analyst 2021, 146, 4180. 34105524 10.1039/d1an00634g

[29] J. Wang , X. Fang , C. Zhang , H. Ji , Q. Pang , X. Li , Z. Luo , Q. Wu , L. Zhang , ACS Appl. Mater. Interfaces 2021, 13, 16118. 33787226 10.1021/acsami.1c02072

[30] Z. Tang , Y. Jun , Y. Lv , Y. Li , Z. Zhang , M. Tao , X. Chen , J. He , L. Zhang , Q. L. Wang , J. Drug Target 2020, 28, 186. 31134823 10.1080/1061186X.2019.1624970

[31] W. Ma , Y. Yang , J. Zhu , W. Jia , T. Zhang , Z. Liu , X. Chen , Y. Lin , Adv. Mater. 2022, 34, 2109609. 10.1002/adma.202109609 35064993

[32] S. Honary , F. Zahir , Trop. J. Pharm. Res. 2013, 12, 255.

[33] Y. Li , W. Hong , H. Zhang , T. T. Zhang , Z. Chen , S. Yuan , P. Peng , M. Xiao , L. Xu , J. Control Release 2020, 317, 232. 31783048 10.1016/j.jconrel.2019.11.031

[34] K. Kumar , P. Moitra , M. Bashir , P. Kondaiah , S. Bhattacharya , Nanoscale 2020, 12, 1067. 31845927 10.1039/c9nr08475d

[35] Y. Jun , Z. Tang , C. Luo , B. Jiang , X. Li , M. Tao , H. Gu , L. Liu , Z. Zhang , S. Sun , K. Han , X. Yu , X. Song , G. Tao , X. Chen , L. Zhang , Y. Gao , Q. L. Wang , ACS Appl. Mater. Interfaces 2020, 12, 47330. 32997489 10.1021/acsami.0c15419

[36] W. Stefan , J. T. Anthony , D. Qin , O. Seiichi , A. Julie , F. D. Harold , W. C. W. Chan , Nat. Rev. Mater. 2016, 1, 16014.

[37] X. Li , E. C. Montague , A. Pollinzi , A. Lofts , T. Hoare , Small 2022, 18, e2104632. 34936204 10.1002/smll.202104632

