# Supplementary material for: Development of a Specific Aptamer‐Modified Nano‐System to Treat Esophageal Squamous Cell Carcinoma
Source: Adv Sci (Weinh). 2024 May 5;11(28):2309084. doi: 10.1002/advs.202309084 (PMC11267304; doi:10.1002/advs.202309084)
Supplement: Supplementary file 1 — Supporting Information [file ADVS-11-2309084-s001.pdf]

ADVANCED SCIENCE
Open Access

Supporting Information

for *Adv. Sci.*, DOI 10.1002/advs.202309084

Development of a Specific Aptamer-Modified Nano-System to Treat Esophageal Squamous Cell Carcinoma

Fei Xie, Jinrong Qiu, Congyong Sun, Lulu Feng, Yali Jun, Chao Luo, Xiamei Guo, Bowei Zhang, Yu Zhou, Yuting Wang, Li Zhang and Qilong Wang**

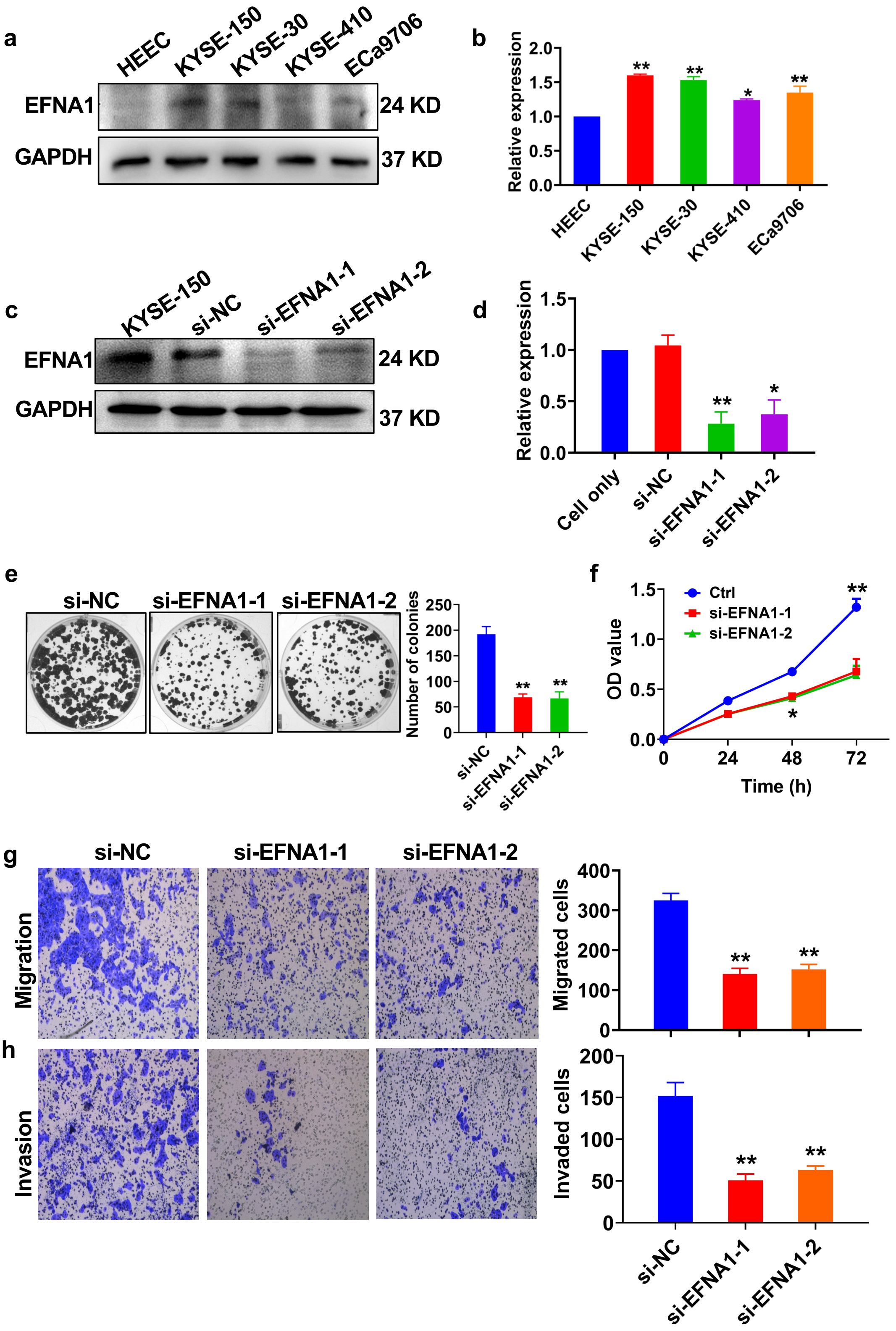

Supplementary Figure 1. Transient transfection of si-EFNA1 inhibits ESCC cell proliferation, migration, and invasion. a, b) Relatively protein expression of EFNA1 by western blotting analysis in human esophageal epithelial cells (HEEC) and various

ESCC cell lines, including KYSE-30, KYSE-150, KYSE-410, and EC9706. c, d) Western blotting showing expression of EFNA1 in KYSE-150 transfected with si-NC and si-EFNA1. e) Effect of EFNA1 on proliferation of si-EFNA1-transfected KYSE150 cells analyzed by colony-formation assay. f) Proliferative ability of KYSE-150 cells transfected with si-NC and si-EFNA1 evaluated using CCK-8 assay. g, h) Effect of EFNA1 on migration and invasion of KYSE-150 cells transfected with si-NC and siEFNA1 tested by Transwell assays, penetrated cells were counted after 48 h of incubation and analyzed by Image J. Data were expressed as mean ± S.D. (n=3). * $p < 0.05$, ** $p < 0.01$, compared with HEEC control or si-NC control group, were regarded as statistically acceptable.

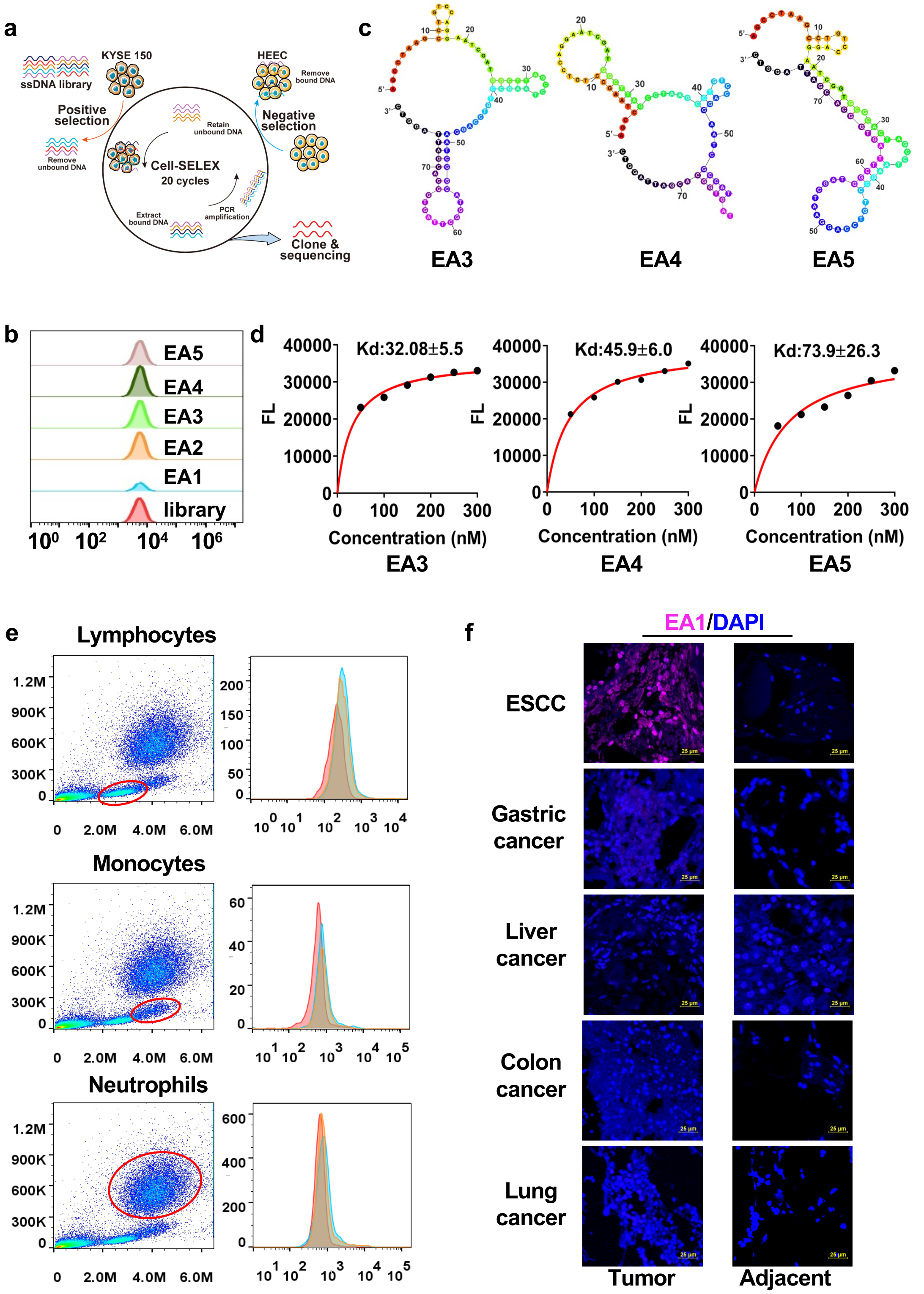

Supplementary Figure 2. Aptamer selection process, affinity, and specificity analysis. a) Schematic representation of cell-SELEX process. ssDNA library was incubated with KYSE-150 cells for positive selection. Bound DNA was extracted and amplified by

PCR for negative selection with HEEC cells. Unbound DNA was retained and used for next-round selection. After repeated selections, saturated library was used for DNA cloning and sequencing. b) Flow cytometry assays of FAM-labeled aptamer candidates to HEEC cells. c) Secondary structures of EA3, EA4 and EA5 predicted by RNAfold. d) Determination of dissociation constant (K_d) of EA3, EA4 and EA5 to KYSE-150 cells using flow cytometry. e) Binding assays of FAM-labeled aptamer EA1 (blue line) and EA2 (yellow line) to human peripheral blood cells by flow cytometry. FAM-labeled random ssDNA (red line) was used as control. Lymphocytes, monocytes, and neutrophils were analyzed. f) Different paired cancer clinical tissues analysis (including ESCC, gastric cancer, liver cancer, colon cancer, lung cancer, and with paired adjacent tissues) by Cy5.5-labeled EA1 using confocal imaging.

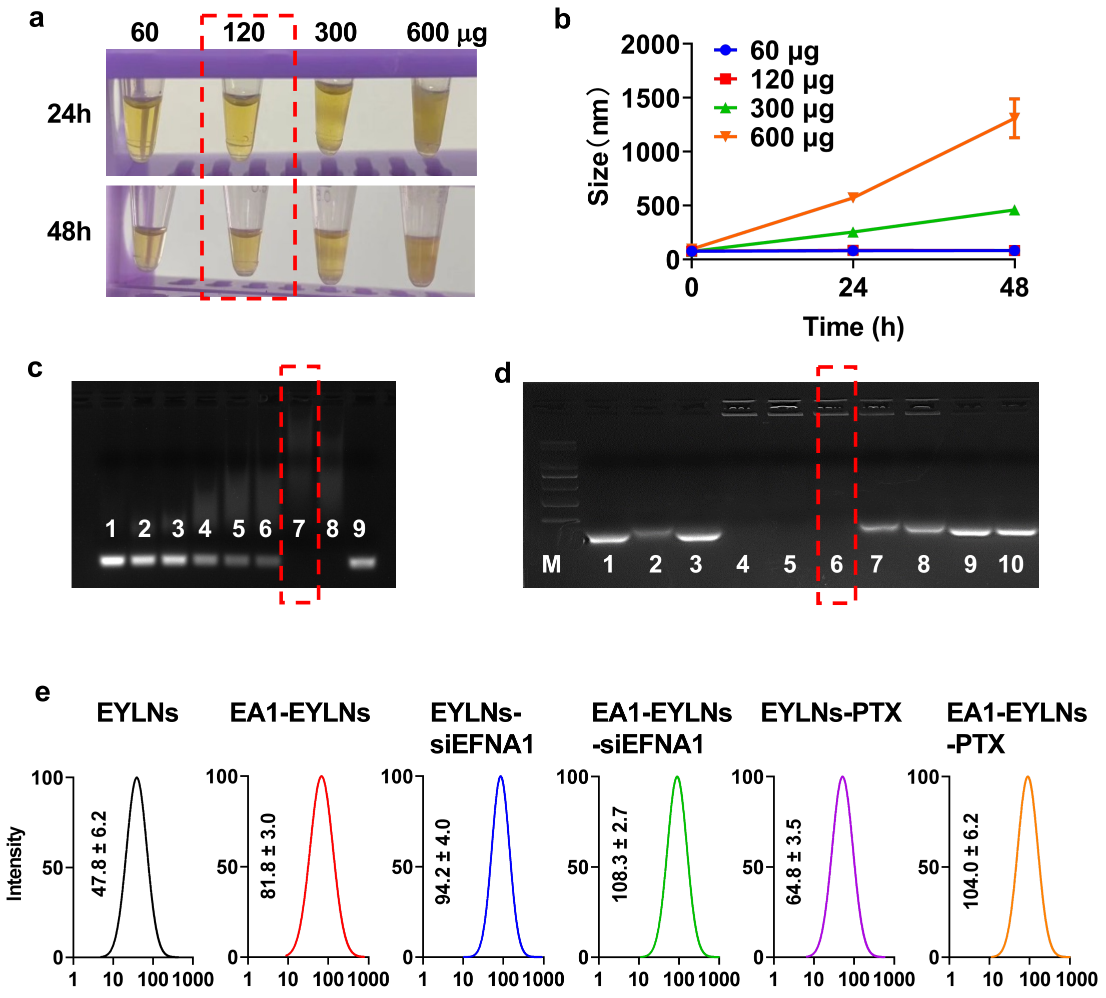

Supplementary Figure 3. Optimization of EYLNs formulation. a) Bright-field images

of different mass (60, 120, 300 and 600 μg) of PTX-loaded EYLNs at 24 h and 48 h. b) Physical stability studies of different mass of PTX-loaded EYLNs. Particle sizes of PTX-loaded EYLNs were determined at 24 h and 48 h. c) 2% Agarose electrophoresis to optimize mass of PEI in EYLNs, ratio was fixed at 3 mg EYLNs: 1.8 nmol siEFNA1: 4.5 nmol EA1, and different mass of PEI was added into EYLNs formulation. Lane1: 20 μg PEI, lane 2: 36 μg PEI, lane 3:45 μg PEI, lane 4: 54 μg PEI, lane 5: 72 μg PEI, lane 6: 90 μg PEI, lane 7: 144 μg, lane 8: 288 μg PEI, lane 9: Free siRNA. d) 2% Agarose electrophoresis to optimize the mass of EA1 in EYLNs; ratio was fixed at 3 mg EYLN: 1.8 nmol siEFNA1: 144 μg PEI, and different mass of EA1 was added into EYLNs. M: DNA ladder, lane 1: Free siEFNA1, lane 2: Free EA1, lane 3: Free EA1 and siEFNA1, lane 4: 3.5 nmol EA1, lane 5: 4 nmol, lane 6: 4.5 nmol, lane 7: 5 nmol, lane 8: 5.5 nmol, lane 9: 6 nmol EA1, lane 10: 6.5 nmol EA1. e) Particle size distribution of EYLNs, EA1-EYLNs, EYLNs-siEFNA1, EA1-EYLNs-siEFNA1, EYLNs-PTX, EA1-EYLNs-PTX.

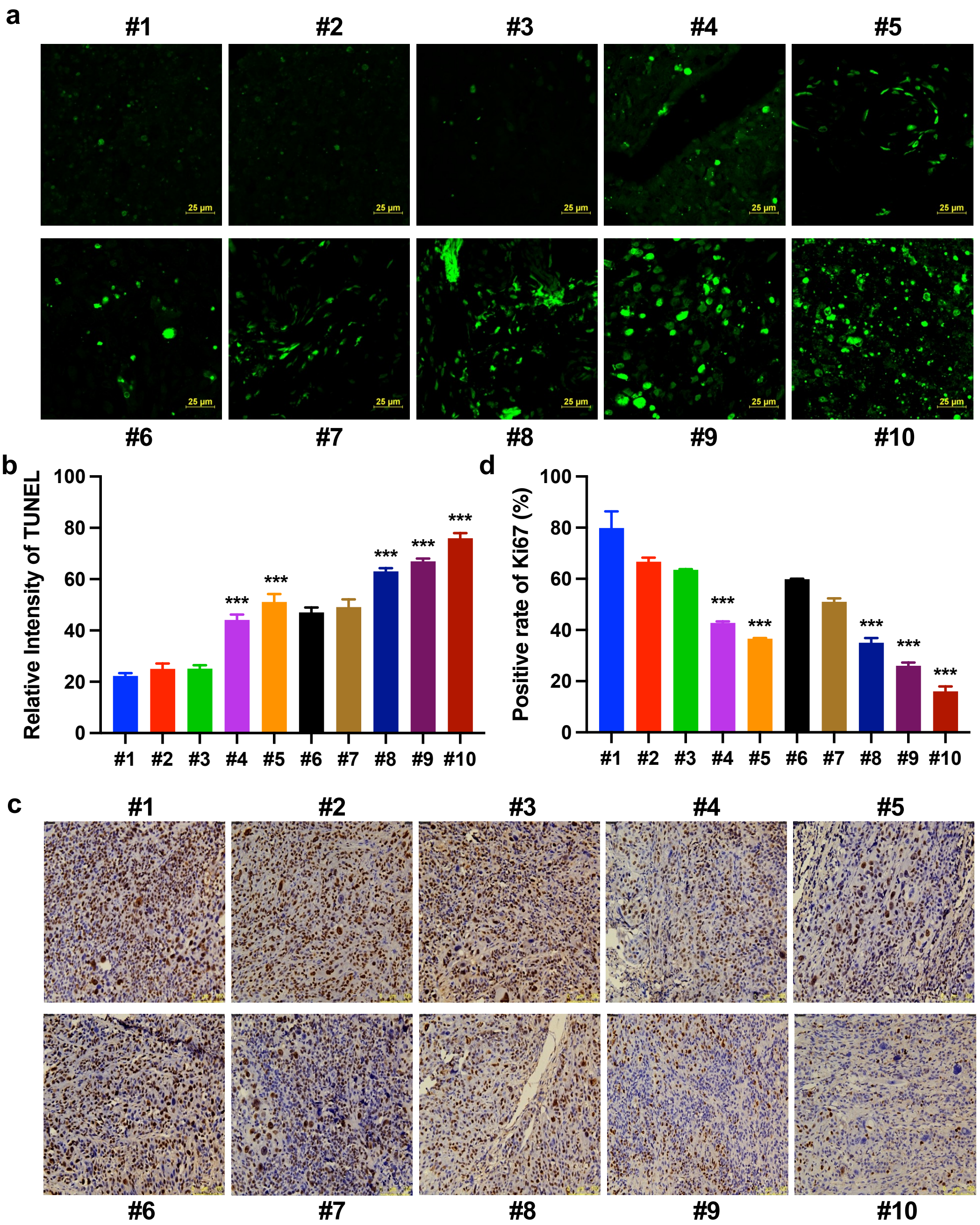

Supplementary Figure 4. Representative a, b) terminal deoxynucleotidyl transferase mediated dUTP nick end labeling (TUNEL) analysis and c, d) IHC of Ki67 in excised tumors after treatment. Treatment includes PBS (#1), EYLNs (5 nmol, #2), EYLNsEA1 (5 nmol, #3), EYLNs-siEFNA1 (5 nmol, #4), EA1-EYLNs-siEFNA1 (5 nmol, #5), PTX (120 nmol, #6), EYLNs-PTX (5 nmol EYLNs with 120 nmol PTX, #7), EA1EYLNs-PTX (#8), EYLNs-PTX/siEFNA1 (#9), and EA1-EYLNs-PTX/siEFNA1 (#10). Scale bar: 25 µm and 100 µm. *** $p < 0.001$, compared with PBS control group,

were regarded as statistically acceptable.

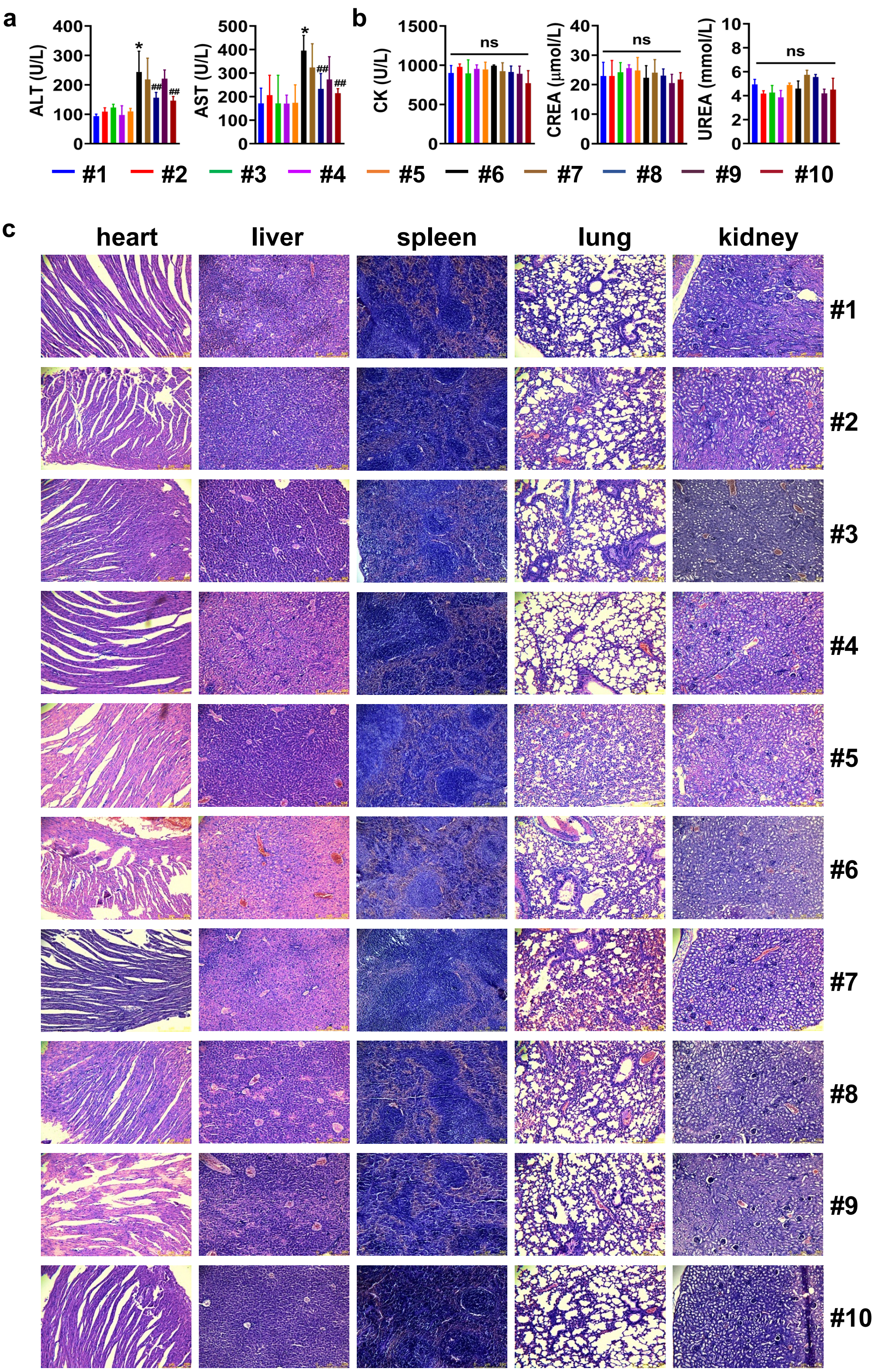

Supplementary Figure 5. In vivo biocompatibility of various NDDS. Serum biochemical indexes a) ALT, AST, b) CK, CREA, and UREA in KYSE-150 tumor

bearing mice. c) Representative photomicrographs of H&E staining of heart, liver, spleen, lung, and kidney tissue sections after various NDDS treatment. Treatment includes PBS (#1), EYLNs (5 nmol, #2), EYLNs-EA1 (5 nmol, #3), EYLNs-siEFNA1 (5 nmol, #4), EA1-EYLNs-siEFNA1 (5 nmol, #5), PTX (120 nmol, #6), EYLNs-PTX (5 nmol EYLNs with 120 nmol PTX, #7), EA1-EYLNs-PTX (#8), EYLNs-PTX/siEFNA1 (#9), and EA1-EYLNs-PTX/siEFNA1 (#10). Scale bar:250 μm.
